# Characterization and Vaccine Potential of Outer Membrane Vesicles Produced by *Haemophilus parasuis*

**DOI:** 10.1371/journal.pone.0149132

**Published:** 2016-03-01

**Authors:** William D. McCaig, Crystal L. Loving, Holly R. Hughes, Susan L. Brockmeier

**Affiliations:** Virus and Prion Diseases Research Unit, National Animal Disease Center, Agricultural Research Service, USDA, Ames, IA, United States of America; Université Paris Descartes, FRANCE

## Abstract

*Haemophilus parasuis* is a Gram-negative bacterium that colonizes the upper respiratory tract of swine and is capable of causing a systemic infection, resulting in high morbidity and mortality. *H*. *parasuis* isolates display a wide range of virulence and virulence factors are largely unknown. Commercial bacterins are often used to vaccinate swine against *H*. *parasuis*, though strain variability and lack of cross-reactivity can make this an ineffective means of protection. Outer membrane vesicles (OMV) are spherical structures naturally released from the membrane of bacteria and OMV are often enriched in toxins, signaling molecules and other bacterial components. Examination of OMV structures has led to identification of virulence factors in a number of bacteria and they have been successfully used as subunit vaccines. We have isolated OMV from both virulent and avirulent strains of *H*. *parasuis*, have examined their protein content and assessed their ability to induce an immune response in the host. Vaccination with purified OMV derived from the virulent *H*. *parasuis* Nagasaki strain provided protection against challenge with a lethal dose of the bacteria.

## Introduction

*Haemophilus parasuis* is a Gram-negative, rod-shaped bacterium belonging to the family *Pasteurellaceae*. This organism is the causative agent of Glässer’s disease in swine, a condition characterized by a severe infection resulting in polyserositis, meningitis and arthritis [1]. *H*. *parasuis* is a member of the normal microbiota in the upper respiratory tract of pigs, even in high health herds [2]. Select strains are capable of spreading to the lungs and systemic sites, resulting in disease and death in the affected host. *H*. *parasuis* is present in all major swine producing countries and results in major economic losses to the swine industry each year [3].

*H*. *parasuis* has been classified into fifteen serotype strains, with ~26% being non-typeable, and isolates exhibiting differences in virulence [4–7]. Strains typically causing disease and death in pigs belong to serotypes 1, 5, 10, 12, 13 and 14, while serotypes 6, 7, 9 and 11 are considered avirulent [4–8]. Strains belonging to serotypes 2, 3, 4, 8 and 15 show an intermediate virulence, causing polyserositis and death in some cases [4]. Protection has been achieved with the use of formalin-inactivated bacterins [9, 10], though heterologous cross-protection against strains from a different serotype can be variable and highly dependent on the vaccine and challenge strains utilized [11, 12]. Vaccination with other members of the *Pasteurellaceae* family, as well as attenuated *H*. *parasuis* strains, can offer some cross-protection against virulent strains [6, 7, 13]. Subunit vaccines composed of bacterial ghosts, DNA, transferrin and other *Haemophilus* proteins have been evaluated, with the efficacy of the vaccines varying [14–18].

Relatively few factors that contribute to the virulence of *H*. *parasuis* have been identified and characterized. A polysaccharide capsule involved in resistance to phagocytosis and complement-mediated killing has been reported [19, 20]. The lipooligosaccharide (LOS) of *H*. *parasuis* is involved in induction of apoptosis, adhesion to certain host cells, and resistance to complement mediated killing [21]. Some strains have been shown to produce cytolethal distending toxins (CDTs) which contribute to serum resistance, as well as host cell adhesion and invasion [22]. The virulence-associated trimeric autotransporters (VTAs), a family of adhesin proteins, have also been found in *Haemophilus*, and a role in delaying phagocytosis has been shown [23, 24]. Two outer membrane porin proteins, OmpP2 and OmpP5, have been identified as potential virulence factors, but a direct role in pathogenesis has yet to be characterized [25]. Additional proteins potentially involved in virulence have been identified, though not completely characterized, including a neuraminidase, IgA protease, and fimbriae [26, 27]. The presence or absence of many of these factors, however, does not always indicate virulence in this organism.

Outer membrane vesicles (OMV) are spherical particles constitutively released from the surface of Gram-negative bacteria [28]. Enriched in virulence factors, signaling molecules and other bacterial components, OMV structures have been isolated from numerous bacteria, their content determined and their role in pathogenesis examined [29, 30]. Multiple functional roles have been proposed for OMV, including secretion of virulence factors, bacterial envelope stress relief, inter- and intracellular communication, gene transfer, biofilm formation and host immune modulation [31, 32]. OMV are capable of delivering their content to host cells, through fusion with the plasma membrane or uptake via endocytic routes [33, 34]. Examination of the host immune response to OMV shows that they are capable of altering production of cytokines, regulating cell-surface molecules and triggering apoptosis [35–38]. Researchers have explored the use of OMV as vaccines against both human and animal pathogens, demonstrating their ability to provide protection against challenge with the parental strain from which they are derived [39–42]. No previous studies of OMV production by *H*. *parasuis* have been reported, though examination of OMV produced by the related human pathogen *H*. *influenza* has led to identification of virulence factors, potential mechanisms for diversion of the host immune response and use as a potential vaccine [35, 40, 43, 44].

The aim of this study was to examine the protein content of OMV from both avirulent (D74, serotype 9) and virulent (Nagasaki, serotype 5) strains of *H*. *parasuis* and examine use of the latter as an acellular subunit vaccine. A number of standard techniques were utilized to accomplish this goal, as experiments of this nature have been successfully applied to a number of other bacteria, though never *Haemophilus parasuis*. Determining the proper growth conditions under which to isolate OMV was the beginning of this process, as media and method of cultivation can have an extreme effect on protein content and yield of OMV. Examining the effect of the purified OMV on host cells, as measured by RT-PCR and cytotoxicity assays, determined the potential host response to a vaccine composed of these structures. Comparison of the protein content between the avirulent and virulent strains identifies potential virulence factors that may be packaged into OMV. The final test was an animal study to examine whether these structures protect the host from infection with *Haemophilus parasuis*.

## Materials and Methods

### Bacterial strains and growth conditions

Media for growth of *H*. *parasuis* strain Nagasaki and D74 was BHI [brain heart infusion powder (BD Biosciences; 37 g/l)], TS [tryptic soy powder (BD Biosciences; 30 g/l)], Casman’s [Casman broth base (HIMEDIA; 29.6 g/l)] supplemented with 10% heat-inactivated horse serum and 0.1mg/ml nicotinamide adenine dinucleotide (NAD^+^). For growth on solid media, Bacto agar (BD Biosciences) was added to 15 g/l. Bacteria streaked on agar plates were incubated at 37°C in the presence of 5% CO_2_. Liquid media was incubated at 37°C in the presence of 5% CO_2_ for 1 h prior to inoculation with bacteria, and cultures were grown in the same conditions. Starter liquid cultures (20 ml) were inoculated from single colonies grown on BHI plates, grown overnight at 200 rpm and diluted 1:20 (in 400 ml BHI) to an OD_600_ of 0.05–0.1 to start day cultures. Day cultures were grown to stationary (OD_600_ 1.2 Nagasaki and 2.1 D74) phase, at 150 rpm, which took approximately 12 h. Agar plates for OMV isolation were inoculated with 50 μl frozen stock, spread to create a lawn and incubated for 48 h at 37°C in the presence of 5% CO_2_. For vaccine challenge studies, bacteria were scraped from multiple BHI plates and resuspended in PBS to a final OD_600_ of 0.42 (1.6 × 10^8^ CFU/ml confirmed by serial dilution).

### Purification of OMV

For isolation of OMV from liquid grown *H*. *parasuis*, cultures were grown to early stationary (OD_600_ 1.2 Nagasaki, 2.1 D74) phase in supplemented BHI (400 ml) in a 1-l flask with baffles. Bacteria were pelleted by centrifugation (Beckman Avanti J-25 centrifuge, JLA 16.250 rotor, 7,500 × *g*, 15 min, 2 spins), and the supernatant collected and passed through a 0.22 μm filter unit (Nalgene) to remove intact bacteria. For isolation of plate grown OMV, 1 g of *H*. *parasuis* was scraped from BHI supplemented agarose with an inoculating loop and suspended in 11 ml sterile phosphate-buffered saline (PBS). Plate grown bacteria were mixed by pipetting and subjected to low speed centrifugation (7,500 × *g*, 15 min each) to pellet intact bacteria. Supernatant was removed and 9 ml of fresh PBS was used to re-suspend the bacterial pellet. Resuspension and collection of supernatant was performed a total of 4 times. Supernatants were pooled and subjected to an additional spin (10,000 × *g*, 15 min). The final supernatant was passed through a 0.22 μm filter unit (Nalgene) to remove intact *H*. *parasuis* cells. Bacterial pellets were subsequently processed to generate whole cell sonicate (described below). Vesicles were pelleted from the supernatant by ultracentrifugation (Beckman Optima XL-100K Ultracentrifuge, SW32 Ti rotor, 110,000 × *g*, 1 h, 4°C). The pelleted OMV were re-suspended in 30 ml 20 mM HEPES (pH 7.5), subjected to an additional ultracentrifugation step (110,000 × *g*, 1 h, 4°C), and resuspended again in 0.5 ml 20 mM HEPES (pH 7.5). The vesicles were then adjusted to 45% (v/v) OptiPrep (Axis-Shield) in a total of 2 ml. Samples were loaded into a 13.2 ml ultracentrifuge tube and lower concentration OptiPrep solutions were layered on top (2 ml 40%, 2 ml 35%, 2 ml 30%, 2 ml 25%, 1 ml 20% and 1 ml 15%). The tubes were centrifuged (Beckman Optima XL-100K Ultracentrifuge, SW41 Ti rotor, 100,000 × *g*, 16 h, 4°C) in a swinging-bucket rotor, and then fractions were collected in 1 ml aliquots from the top of the gradient. Fractions were examined by SDS-PAGE (sodium dodecyl sulfate-polyacrylamide gel electrophoresis, 12% gel) and Coomassie blue staining for protein content. Adjacent fractions from the same strain and growth conditions with similar protein profiles were combined, diluted with 20 mM HEPES (pH 7.5, final volume 30 ml) and pelleted via ultracentrifugation (110,000 × *g*, 1 h, 4°C). Recovered pellets were resuspended in 20 mM HEPES (pH 7.5) and plated on supplemented BHI agar to verify sterility. Concentrations of OMV were determined using the bicinchoninic acid (BCA) protein assay (Pierce), according to manufacturer’s instructions, with the addition of 2% SDS [45].

### Preparation of whole cell sonicate and lysate of *H*. *parasuis*

Bacterial pellets collected during OMV purification were suspended in 50 ml 50 mM Tris-HCl (pH 7.4), 150 mM NaCl, 10 mM EDTA containing 1 tablet of Complete, EDTA-free protease inhibitor cocktail (Roche Diagnostics). Suspensions were divided into 10 ml aliquots and heated to 56°C for 30 minutes. Suspensions were cooled on ice and then sonicated (Misonix Sonicator 3000; power level 5) in an ice water bath, 15 s on and 15 s off, for 4 min. Sonicated bacterial suspensions were centrifuged (7,500 × *g*, 15 min, 4°C) to remove intact bacterial cells. The supernatants were pooled and then ultracentrifuged (110,000 × *g*, 1 h, 4°C) to pellet membranes [46]. The membrane pellet was resuspended in 1ml of 20 mM HEPES (pH 7.5), and then adjusted to 45% (vol/vol) OptiPrep (Axis-Shield) in a total volume of 2 ml and purified as described above.

*H*. *parasuis* sonicate for coating ELISA plates was generated from Nagasaki strain grown in supplemented BHI liquid cultures grown to early stationary phase. *H*. *parasuis* was pelleted and suspended in 50 ml 50 mM Tris-HCl (pH 7.4), 150 mM NaCl, 10 mM EDTA. Suspended bacteria were heat-treated to 56°C for 30 min, cooled on ice and then sonicated as above. Sonicated bacterial suspensions were centrifuged (7,500 × *g*, 15 min, 4°C) to remove intact bacterial cells and supernatant was collected as sonicate material. Sonicate was plated on supplemented BHI agar, confirmed to be sterile and the concentration was determined using the bicinchoninic acid (BCA) protein assay (Pierce), according to manufacturer’s instructions, with the addition of 2% sodium dodecyl sulfate.

Whole cell lysates of Nagasaki and D74 were generated by growth in liquid supplemented BHI media to OD_600_ of 0.5–0.6. One ml of culture was centrifuged (Eppendorf 5424R centrifuge, FA-45-24-11 rotor, 7,500 × *g*, 5 minutes, 4°C) to pellet bacteria and supernatant was discarded. Bacterial pellets were suspended in 0.1 ml 6M Urea and boiled at 100°C for 10 min. Lysates were aliquoted and stored at -20°C until SDS-PAGE analysis was performed.

### Transmission electron microscopy (TEM)

Samples were adsorbed to polyvinyl formal-carbon-coated grids (EMS) for 2 min, fixed with 1% gluteraldehyde for 1 min, washed twice with PBS and twice with water, and then negatively stained with 0.5% phosphotungstic acid for 20 s. All grids were viewed in a FEI Tecnai12 BioTwinG^2^ electron microscope at 80 kV accelerating voltage, and images were obtained using a Hamamatsu ORCA HR camera and compiled using ImageJ software (NIH) [47].

### Generation of immune sera used for Western Blots to characterize protein profile patterns of OMV of *H*. *parasuis*

For generation of formalin killed vaccine, *H*. *parasuis* strain Nagasaki bacteria were scraped from Casman’s plates and resuspended in PBS to a final OD_600_ of 0.42, an approximate CFU value of 1× 10^9^. 125 μl of 10% formalin was added to 50 ml bacterial suspension and rocked over night at room temperature. 100 μl of the bacterial suspension was plated on supplemented Casman’s agar to verify sterility. The bacterial suspension was centrifuged, washed with PBS and resuspended in 50 ml PBS. A vaccine consisting of 80% bacterial suspension and 20% adjuvant (Emulsigen-D, MVP laboratories) was used to vaccinate pigs. Six, 8 week old piglets farrowed from caesarean-derived, colostrum-deprived pigs bred and raised on site were vaccinated intramuscularly with 2 ml of the above vaccine preparation and boosted 21 days later with an additional 2 ml of vaccine. Blood was drawn prior to vaccination and 27 days after vaccine boost; serum was saved and stored at -80°C until used for Western Blots. Sera from all six pigs was pooled and utilized at the indicated concentration.

### Protease accessibility assay

Purified OMV were left untreated or treated with proteinase K (200 μg/ml), SDS (0.02%), or proteinase K plus SDS for 1 h at 37°C. Phenylmethanesulfonyl fluoride (PMSF, 10 mM) was added to inhibit proteases (including proteinase K) and samples were heated at 100°C for 10 min in SDS sample buffer for subsequent SDS-PAGE analysis. For immunoblotting, proteins separated by SDS-PAGE were transferred to a nitrocellulose membrane using an iBlot system (Invitrogen) and probed with porcine sera (1:500 dilution). Immunoblots were developed with HRP-conjugated secondary goat anti-swine IgG (Kirkegaard and Perry Laboratories, 0.25 μg/ml) and the Pierce ECL 2 Western Blotting substrate (Thermo Scientific).

### Mass spectrometry

Purified OMV were analyzed by mass spectrometry using the MudPIT (multidimensional protein identification technology) method [48]. All MS/MS samples were analyzed using Sequest (Thermo Fisher Scientific, San Jose, CA, USA; version 1.4.1.14). Sequest was set up to search a Nagasaki (26418 entries) or D74 (26352) database assuming the digestion enzyme trypsin. Sequest was searched with a fragment ion mass tolerance of 0.80 Da and a parent ion tolerance of 10.0 PPM. Carbamidomethyl of cysteine was specified in Sequest as a fixed modification. Oxidation of methionine was specified in Sequest as a variable modification. Scaffold (version Scaffold_4.4.1.1, Proteome Software Inc., Portland, OR) was used to validate MS/MS based peptide and protein identifications. Peptide identifications were accepted if they could be established at greater than 95.0% probability by the Scaffold Local FDR algorithm. Protein identifications were accepted if they could be established at greater than 99.0% probability and contained at least 2 identified peptides. Two independent experiments were performed for each strain and growth condition, each sample was analyzed twice. Only proteins identified in both biological samples and both replicate mass spectometry experiments under similar growth conditions were considered as vesicle-associated. For the liquid grown sonicate controls, one biological sample from each strain was analyzed twice and only proteins appearing in both analysis were considered associated with the purified sonicate. A normalized spectral abundance factor (NSAF) was used to quantify the relative amounts of individual proteins in each sample and values were averaged between the same biological and experimental samples [49].

Predicted localization of *H*. *parasuis* proteins was determined using the pSORTb v 3.0 [50] software in conjunction with Cello2Go [51]. Comparison of individual *H*. *parasuis* Nagasaki proteins to D74, or to the OMV-associated content of other bacteria was accomplished using the Basic Local Alignment Search Tool [52].

### Preparation of porcine alveolar macrophages

Porcine alveolar macrophages (AMac) were isolated from healthy conventional pigs as previously described [53]. Briefly, lungs were lavaged with 300 ml of PBS. Collected lavage was centrifuged at 400 × g for 15 min, cells washed once with PBS, and cells resuspended in supplemented medium [RPMI 1640, 5% swine sera, 5 mM HEPES, 1 mM L-glutamine, antibiotic-antimycotic, and 50 μg/ml gentamicin (Invitrogen Life Technologies)]. Cells were cultured in 150 × 15-mm petri dishes for 2 h at 37°C in 5% CO_2_. Non-adherent cells were removed and adherent AMac harvested with a cell scraper, collected, washed once, and counted on a Z2 Coulter Particle Count and Size Analyzer (Beckman Coulter). AMac were seeded at 2.5 × 10^5^ cells per well in a 48-well flat-bottom plate with a final volume of 0.5 ml for studies (RT-PCR and cytotoxicity). Cells were allowed to adhere for an additional 2 hours before stimulation. Non-stimulated cells were included for each biological sample as a control.

### Cytotoxicity assays

AMac isolated from five pigs were tested for LDH release after stimulation with 1 μg each of Nagasaki liquid OMV, Nagasaki plate OMV, Nagasaki liquid sonicate, D74 liquid OMV and 10 μg *E*. *coli* 0111:B4 LPS (Invitrogen) LPS as a control. Purified OMV, whole cell sonicate or LPS were resuspended in room temperature supplemented media (RPMI 1640, 5% swine sera, 5mM HEPES, 1mM l-glutamine) at indicated concentrations. The supernatant was removed from AMac previously seeded into 48-well plates, and 500 μl of media with appropriate sample (OMV, sonicate, LPS) was added to the wells. Cells were incubated at 37°C, 5% CO_2_ for 18 h, supernatants were collected and clarified (200 × g, 5 minutes). A lactate dehydrogenase (LDH) assay (CytoTox 96 Non-Radioactive Cytotoxicity Assay, Promega) was performed according to manufacturer’s instructions. Background LDH release was measured in medium from cells stimulated with only media, while total LDH release (= 100% cytotoxicity) was measured from unstimulated cells that were lysed by freezing and thawing. The percentage of LDH release was calculated by subtracting the background LDH release value from the LDH release value of the treated cells, and this number was then divided by the total LDH release value and multiplied by 100.

### AMac stimulation

AMac were stimulated with purified OMV (1 μg), whole cell sonicate (1 μg), live *H*. *parasuis* (MOI 2:1) or *E*. *coli* 0111:B4 LPS (10 μg/ml) (Invitrogen). After 18 h incubation, media was collected and cells were lysed on the culture plate with TRI reagent (Ambion). RNA was isolated using a MagMax Express (Life Technologies) and cDNA was synthesized using a SuperScript VILO cDNA kit (Invitrogen). SYBR Green-based real-time PCR was conducted for various mRNA targets using SYBR Green Master mix (Life Technologies) following manufacturers’ protocol. All samples were run in duplicate. Levels of mRNA were calculated using the threshold cycle 2^-ΔΔCt^ method, which expresses mRNA in treated cells relative to non-stimulated cells after normalizing to β-actin [54]. Primers used were previously reported [55] and PCR products were < 100 bp in size.

### Vaccination and challenge experiments

Twelve, 8–9 week old piglets farrowed from caesarean-derived, colostrum-deprived pigs bred and raised on site were vaccinated intramuscularly with 25 μg (protein concentration) Nagasaki liquid derived OMV or sonicate (6 pigs each) in 1 ml PBS, boosted 21 days later with an additional 25 μg and intranasally challenged 14 days after the boost with 3 × 10^8^ colony-forming units (CFU) of BHI plate grown *H*. *parasuis* Nagasaki. Four piglets served as non-vaccinated, challenged controls. Pigs were observed for clinical signs of systemic disease, including lameness, lethargy, or neurological signs, and were humanely euthanized when systemic clinical signs appeared or 15 days post challenge if clinical signs were not observed, at which time samples were collected and plated for bacterial burden (nasal, hock joint, serosa, cerebral spinal fluid, lung lavage). Blood was drawn prior to vaccination, bacterial challenge and at necropsy; serum was saved and stored at -80°C until used for Western Blots or ELISAs.

### *H. parasuis* ELISA

Nagasaki sonicate was diluted in coating buffer (0.1M Carbonate-bicarbonate buffer, pH 9.6) to a final protein concentration of 0.1 μg/ml per well of an Immulon-2 ELISA plate (Nunc), incubated overnight at 4°C, washed three times with PBS-Tween (0.05%), and blocked with 1% bovine serum albumin in PBS for 1 h at room temperature. Blocked plates were washed as above, and serum from each pig was diluted 1:250 in 1% bovine serum albumin in PBS and titrated 2-fold in duplicate, and incubated at room temperature for 1 h. Gnotobiotic pig sera was used as a control. Wells were washed and 0.1 μg/ml HRP-conjugated secondary goat anti-swine IgG (Kirkegaard and Perry Laboratories) was added to each well and incubated at room temperature for 1 h, washed and ABTS peroxide substrate solution (Kirkegaard and Perry Laboratories) was added to each well and incubated for 30 min in the dark. The reaction was stopped by addition of Stop Solution (Kirkegaard and Perry Laboratories) and absorbance was read at 405 nm. The resulting absorbance values were modeled as a nonlinear function of the Log_10_ dilution using GraphPad Prism (GraphPad Software, Inc.) log (agonist) vs. response-variable slope four-parameter logistic model. Endpoints were interpolated by using twice the average absorbance value of the gnotobiotic control for all dilutions from each plate as the cutoff.

### Statistical analysis

Cytotoxicity results were analyzed for significance using data obtained from five total animals in two independent experiments. Real-time PCR results were analyzed for significance using data obtained from seven separate animals in two independent experiments. Probability (*P*) values were calculated by one-way analysis of variance and Bonferroni’s multiple-comparison post-test against the negative control value. The log-rank test was used to calculate the *P* value for the vaccine challenge experiments, using the combined survival data. A paired t-test was used to calculate the significance between endpoint titers of pre and post vaccination sera from each experimental group. Statistical calculations were performed using Prism 6.02 (GraphPad Software Inc., La Jolla, CA). *P* values < 0.05 were considered significant.

### Ethics statement

Animal studies were conducted in accordance with the recommendations in the Guide for the Care and Use of Laboratory Animals of the National Institutes of Health. The animal experiments were approved by the USDA-National Animal Disease Center's Institutional Animal Care and Use Committee. During the course of the animal experiments in which pigs were challenged with virulent *H*. *parasuis*, pigs were examined for clinical signs approximately every 4 hours except for an 8 hour overnight period and any pig showing signs of systemic disease, such as neurologic involvement, severe lameness, or depression that resulted in recumbency with reluctance to stand, were humanely euthanized with an overdose of barbiturate.

## Results

### *H*. *parasuis* OMV Production

*Haemophilus parasuis* can be cultivated in a number of complex liquid media supplemented with nicotinamide adenine dinucleotide (NAD) and heat-inactivated serum. We examined the growth of this organism with a number of liquid and solid media (BHI, tryptic soy (TS), and Casman’s) before choosing BHI for our experiments. When grown in BHI liquid media, D74 and Nagasaki strains reached maximum OD_600_ values at approximately 2.1 and 1.3, with maximum CFU values of 2.5 × 10^9^/ml (D74) and 9 × 10^9^/ml (Nagasaki) (S1 Fig and Table 1). We observed an increase in extracellular structures via EM, particularly with the Nagasaki strain, when *H*. *parasuis* was grown on BHI plates, in comparison to Casman's and TS plates (S2 Fig). When grown on solid media, colonies appeared on BHI agar within 24 h and bacterial lawns were confluent within 48 h. For these reasons, we chose stationary phase liquid cultures (12 hours) using BHI media, as well as growth on BHI plates for 48 h, as conditions for examination of OMV production in strain D74 and Nagasaki. Additionally, we created a whole cell sonicate as a control condition where vesicles form spontaneously as a result of cell lysis. Bacterial preps from which OMV were isolated were diluted and plated to determine CFU and protein concentration for OMV was determined prior to density gradient separation (Table 1).

**Table 1:** *H*. *parasuis* CFU and OMV totals. Experimental growth conditions, CFU and OMV yields.

Strain (growth condition)	Abbreviation	CFU/ml (average)	Volume (culture or PBS)	CFU total (average)	Raw OMV total (average)	Purified total (average)
Nagasaki (plate)	NPO	3.85 × 10^9^	46 ml	1.77 × 10^11^	2.9 mg	2.0 mg
Nagasaki (liquid)	NLO	8.98 × 10^9^	380 ml	3.41 × 10^12^	2.6 mg	0.7 mg
D74 (plate)	DPO	3.29 × 10^9^	46 ml	1.85 × 10^11^	2.4 mg	1.5 mg
D74 (liquid)	DLO	1.68 × 10^9^	380 ml	6.39 × 10^11^	3.4 mg	1.0 mg

**Fig 1:**
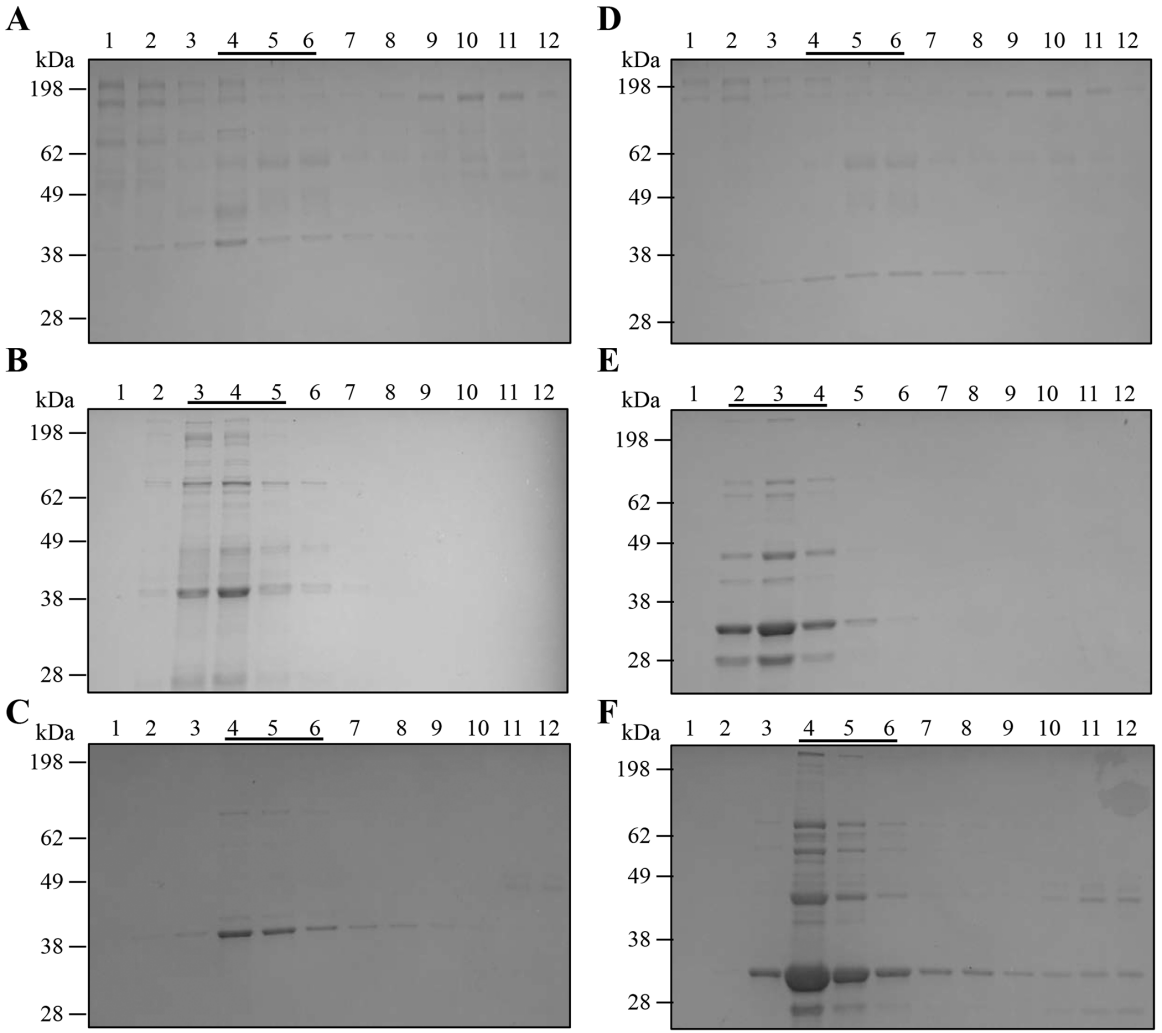
Density gradient purification of vesicles. A. D74 Liquid OMV density fractions 1–12, B. D74 Plate OMV density fractions 1–12, C. D74 Liquid Sonicate density fractions 1–12, D. Nagasaki Liquid OMV density fractions 1–12, E. Nagasaki Plate OMV density fractions 1–12, F. Nagasaki Liquid Sonicate density fractions 1–12. Iodixinol concentrations by lane, 1 = 15%, 2 = 20%, 3–4 = 25%, 5–6 = 30%, 7–8 = 35%, 9–10 = 40%, 11–12 = 45%. Bars indicate fractions combined for OMV recovery.

**Fig 2:**
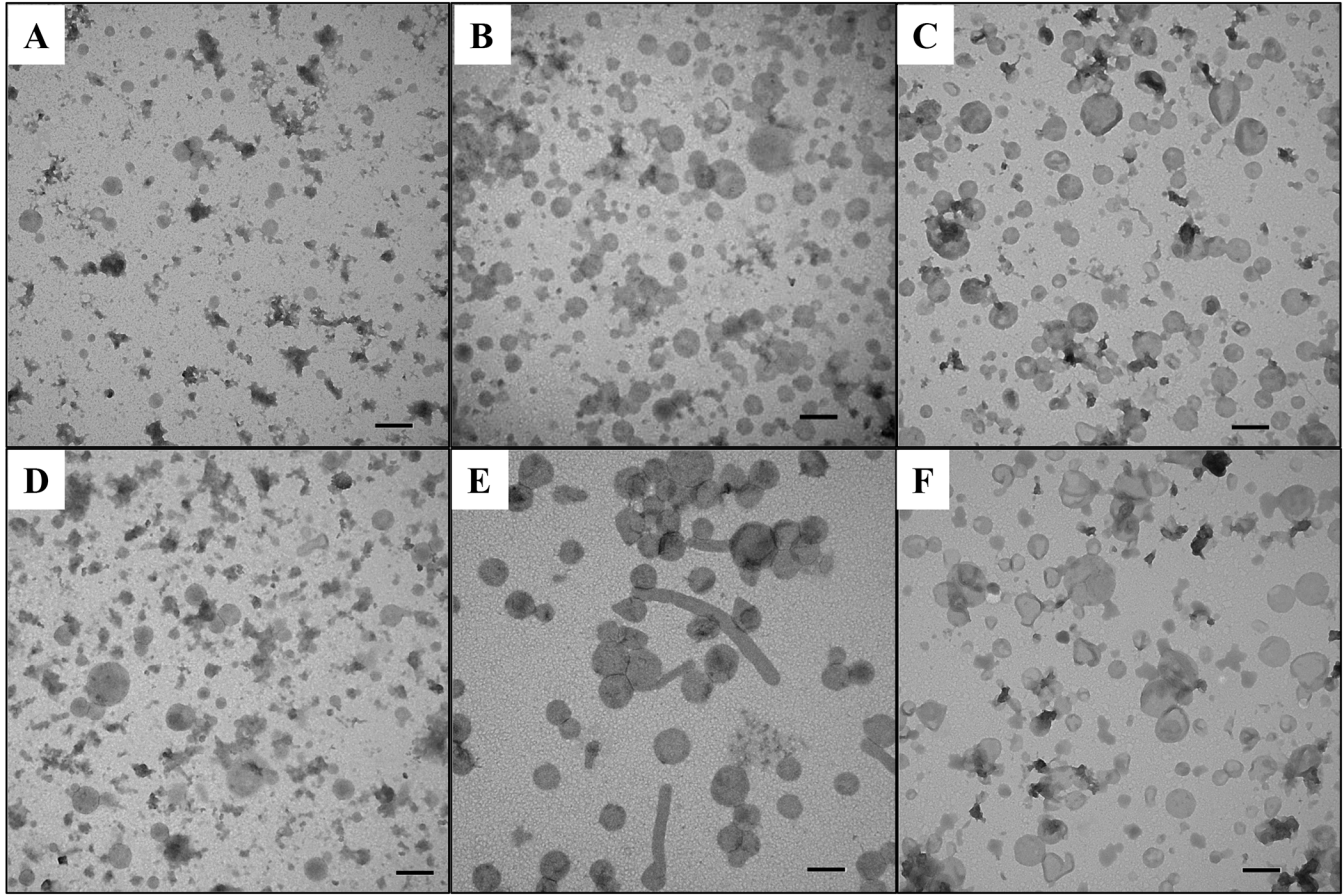
Transmission electron microscopy of purified OMV and sonicate samples. A. D74 liquid OMV, B. D74 plate OMV, C. D74 liquid Sonicate, D. Nagasaki liquid OMV, E. Nagasaki plate OMV, F. Nagasaki liquid Sonicate, Scale Bar = 200nm.

OMV pellets were further purified by flotation through a discontinuous iodixanol (OptiPrep) density gradient. Fractions were collected from each gradient, a portion of each fraction was separated by SDS-PAGE and gels were stained to visualize protein content (Fig 1). SDS-PAGE results showed the majority of proteins from plate isolated OMV migrated to the 15–20% (Lanes 2–4 Nagasaki plate, Fig 1E) or 20–25% (Lanes 3–5 D74 plate, Fig 1B) fractions of the density gradient. Protein in the whole cell sonicate (for both strains) was observed primarily in the 25–30% range of the density gradient (Lanes 4–6, Fig 1C and 1F). For liquid isolated OMV, protein was detected throughout all of the density fractions (Fig 1A and 1D). We relied on similar protein banding patterns observed in neighboring fractions (Fig 1A and 1D, Lanes 2–3 and 4–6) to determine which fractions to combine and recover for further analysis of OMV. Recovered fractions were also tested for total protein using BCA and quality of recovered vesicles verified by transmission electron microscopy (TEM, Fig 2). As can be seen in the micrographs, recovered OMV samples isolated from liquid grown cultures (Fig 2A and 2D) contain typical round structures 50–200 nm in diameter, as well as some debris which may be capsular material or aggregated proteins. OMV samples isolated from plate grown cultures (Fig 2B and 2E) contain some larger spherical structures (> 200 nm), in addition to the typical 50–200 nm OMV. OMV samples isolated from plate grown Nagasaki (Fig 2E) also contain a number of tube-like structures that are several micron long. Purified sonicate samples isolated from liquid grown cultures (Fig 2C and 2F) contain a mix of structures, including several large (> 200 nm), round spherical objects. Attempts at recovering the 15–20% fractions from liquid isolated OMV (Fig 1A and 1D, Lanes 2–3) resulted in minimal protein yields and TEM analysis showed a reduced number of spherical structures (data not shown). For the liquid grown D74 and Nagasaki OMV and sonicate gradients, fractions 4, 5 and 6 were recovered and used for further analysis (Fig 1A, 1C, 1D and 1F). For the plate grown D74 OMV gradient, fractions 3, 4 and 5 were recovered and used for further analysis (Fig 1B). For the plate grown Nagasaki OMV gradient, fractions 2, 3 and 4 were recovered and used for further analysis (Fig 1E).

### Characterization of *H*. *parasuis* OMV

**Fig 3:**
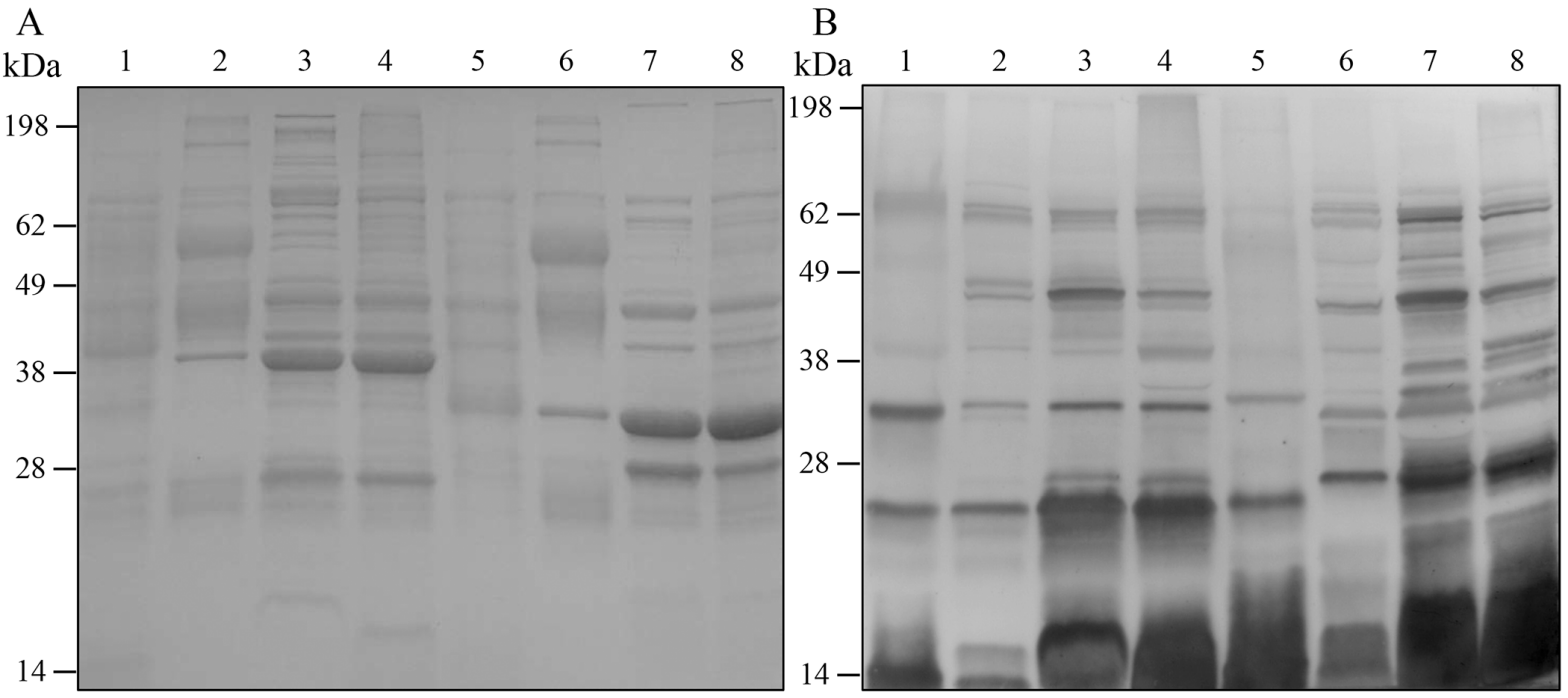
Protein profiles and Western blot of *H*. *parasuis* samples. A. SDS-PAGE of D74 whole cell lysate (Lane 1), D74 liquid OMV (Lane 2), D74 plate OMV (Lane 3), D74 sonicate (Lane 4), Nagasaki whole cell lysate (Lane 5), Nagasaki liquid OMV (Lane 6), Nagasaki plate OMV (Lane 7), Nagasaki plate sonicate (Lane 8) (20 μg/well). B. Same wells and concentration as A, Western blot probed with sera from Nagasaki bacterin immunized pigs (1:1K dilution).

To evaluate the abundance and profile patterns of OMV-associated proteins, purified OMV were separated by SDS-PAGE and stained or transferred to membrane and incubated with sera from Nagasaki bacterin immunized pigs (Fig 3). As a control, we included a whole cell lysate generated from liquid grown bacteria (Fig 3A and 3B, lanes 1 and 5), as well as density purified whole cell sonicate generated from plate grown bacteria (Fig 3A and 3B, lanes 4 and 8). Sonicate generated from liquid grown bacteria had similar profiles to that of plate grown bacteria and was not included (data not shown). We observed different protein profiles when we compared OMV isolated from liquid and plate grown bacteria (Fig 3A lanes 2 and 3, 6 and 7) or to vesicles generated by sonication, even when prepared from the same *H*. *parasuis* strain. OMV-associated proteins probed with *H*. *parasuis* immune sera showed differences in profiles between purified OMV and sonicate or lysate, as well as between the two strains (Fig 3B). Sera isolated from pigs prior to bacterin immunization showed minimal reactivity to the same *H*. *parasuis* OMV preparations (data not shown). There were similarities in protein banding patterns between purified OMV and sonicate, however the differences in protein abundance and immunoreactive protein patterns suggest that OMV content is not solely due to spontaneous vesicle formation by cell lysis.

**Fig 4:**
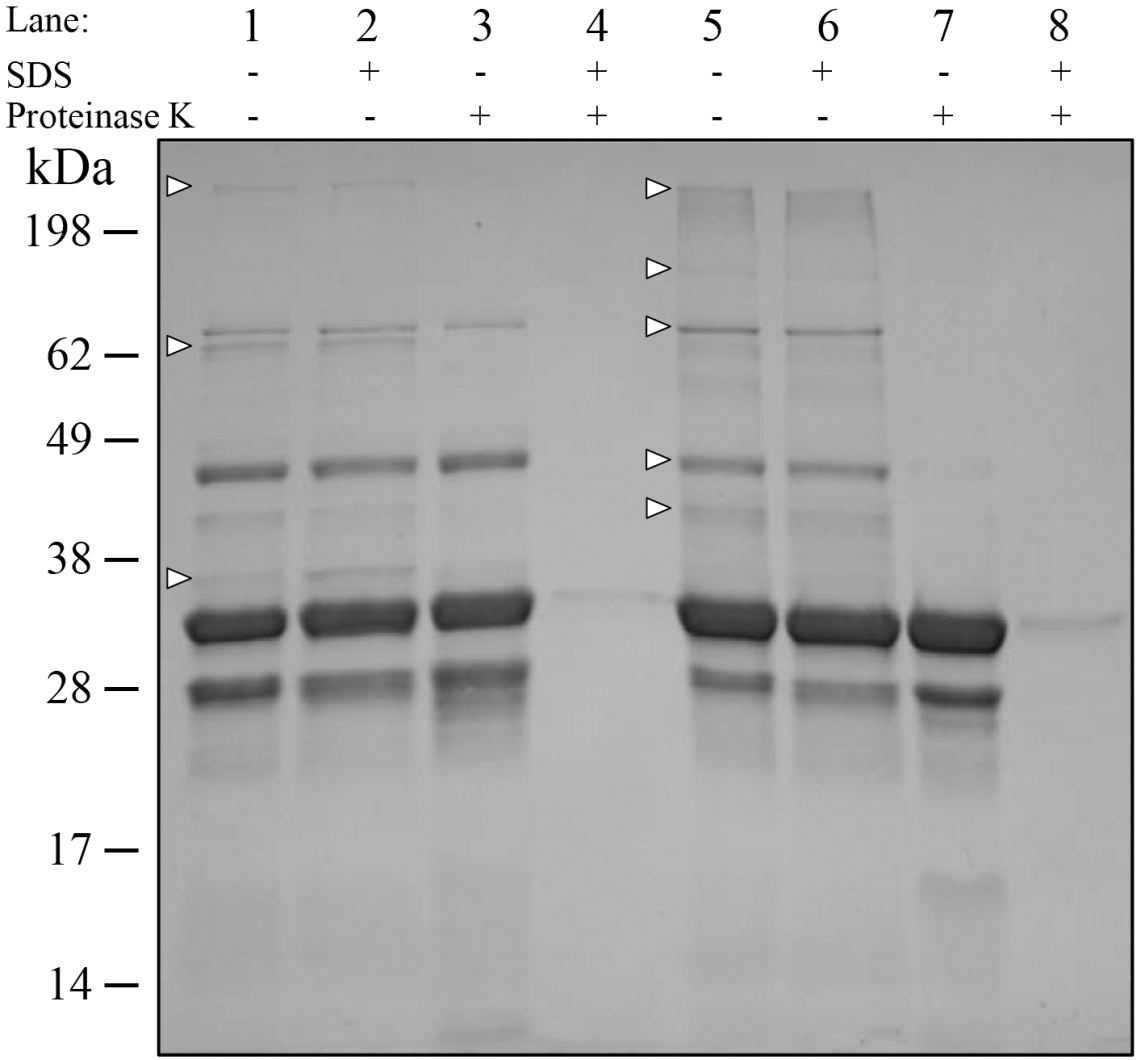
Protease accessibility assay on Nagasaki OMV and sonicate. 10 μg purified Nagasaki plate OMV (lanes 1–4) and Nagasaki plate Sonicate (lanes 5–8), untreated (lane 1 & 5), 0.02% SDS treated (lane 2 & 6), 200 μg/ml proteinase K (PK) treated (lane 3 & 7), 0.02% SDS and proteinase K treated (lane 4 & 8) SDS-PAGE stained with Coomassie Blue, arrowheads indicate proteins missing after PK treatment.

To further verify *H*. *parasuis* OMV exhibited similar properties to OMV described for other bacterial organisms, we performed a protease accessibility assay, which tests the ability of OMV structures to protect proteins against degradation by extracellular proteases [56]. Purified plate isolated OMV and sonicate were incubated with proteinase K (with or without SDS) and protein profiles then examined by SDS-PAGE (Fig 4). The addition of SDS to vesicles had no effect on overall protein content (Fig 4, lanes 2 and 6), however, incubation with proteinase K alone showed degradation of some surface exposed proteins (Fig 4, lanes 3 and 7), an effect which was more pronounced in the sonicate than in the OMV (Fig 4. compare lane 5 to 7, white arrows). When samples were incubated with proteinase K in the presence of SDS, we observed degradation of nearly every protein, regardless of the method used to prepare samples (Fig 4, lanes 4 and 8).

**Fig 5:**
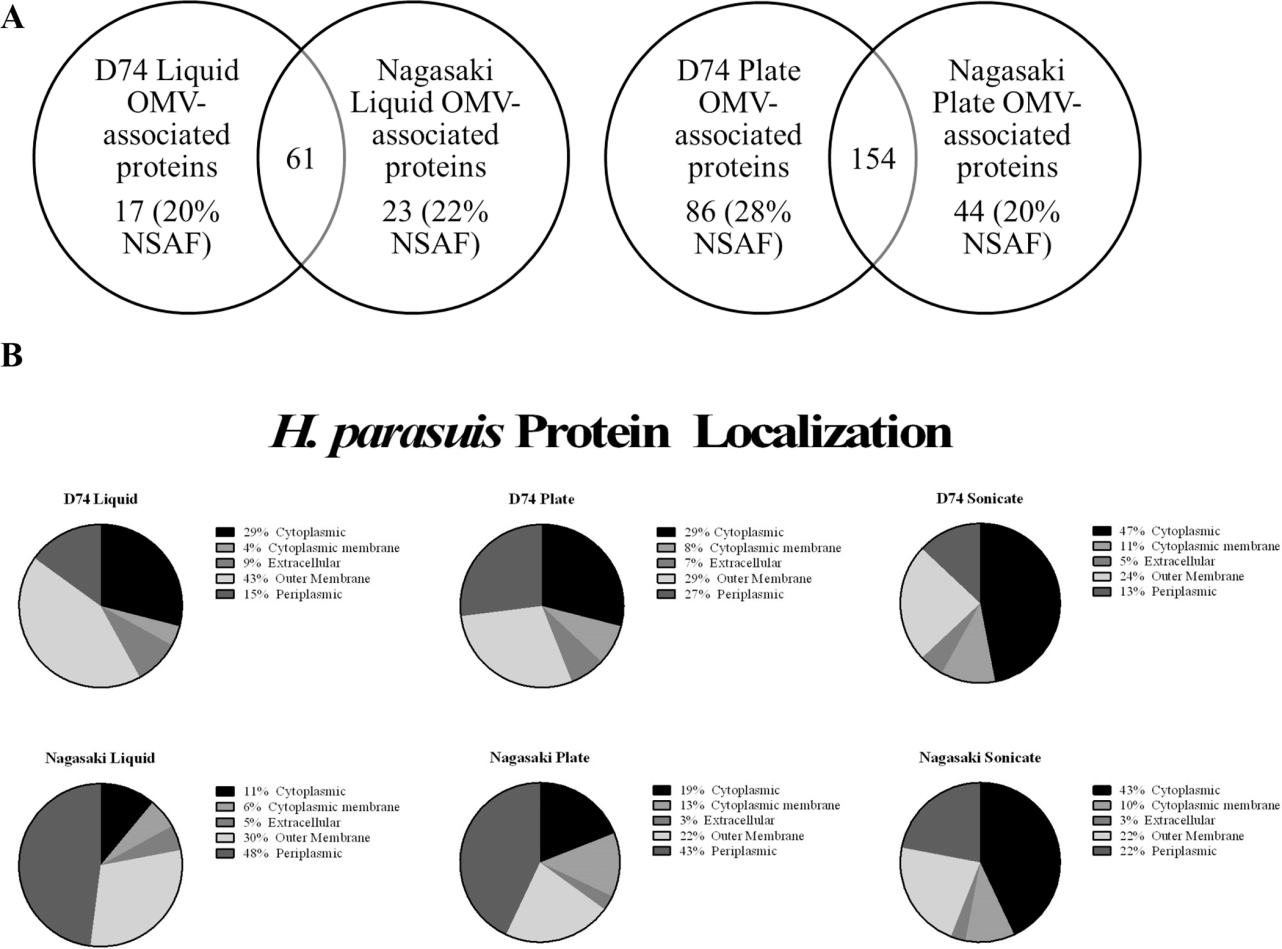
*H*. *parasuis* purified OMV and sonicate protein localization. A. Unique and shared proteins from liquid and plate isolated OMV, B. Protein localization as predicted by the pSORTb 3.0 and CELLO2GO programs. NSAF = Normalized Spectral Abundance Factor.

Purified vesicles were subjected to mass spectrometry using the MudPIT method to identify proteins associated with OMV. MudPIT analysis identified 84 proteins associated with liquid-derived OMV from strain D74 and 78 proteins from strain Nagasaki, with a combined identification of 101 unique OMV-associated proteins (S1 Table and Fig 5A). Analysis of plate isolated OMV identified 240 proteins associated with OMV from D74 and 198 proteins associated with OMV from Nagasaki, with a combined identification of 284 unique OMV-associated proteins (S2 Table and Fig 5A). Analysis of the purified sonicate controls identified 416 proteins associated with strain D74 and 360 proteins associated with strain Nagasaki. The 19 most abundant proteins (NSAF ≥ 0.02 for at least one strain/growth method) are listed in Table 2.

**Table 2:** NSAF values of top proteins from liquid and plate isolated OMV. Top proteins (NSAF> = 0.02 for at least one experimental condition) from Nagasaki plate isolated OMV (NPO), D74 plate isolated OMV (DPO), Nagasaki liquid isolated OMV (NLO) and D74 liquid isolated OMV (DLO).

Nagasaki Locus	D74 Locus	Protein Description	Mol wt (kDa)	NPO NSAF	DPO NSAF	NLO NSAF	DLO NSAF	Localization	Similarity	Ref.
HPSNAG_0042	HPSD74_0020	peptidoglycan-associated lipoprotein	38	0.0297	0.0116	0.0288	0.0233	O	89%	[70, 71]
HPSNAG_2308	HPSD74_2216	outer membrane protein P5	39	0.0276	0.0191	0.0218	0.0345	O	81%	[43, 70, 71]
HPSNAG_1095	HPSD74_1165	heme-binding protein A	59	0.0270	0.0162	0.0135	0.0124	P	96%	[43, 72]
HPSNAG_1298	HPSD74_1320	L-cystine-binding protein tcyA	28	0.0265	0.0239	0.0055	0.0125	P	98%	[43]
HPSNAG_0439	HPSD74_0534	D-galactose-binding periplasmic protein	35	0.0230	0.0076	0.0244	0.0061	P	96%	[43]
HPSNAG_0730	HPSD74_0873	superoxide dismutase	19	0.0192	0	0.0259	0	P	88%	[70]
HPSNAG_1330	HPSD74_1964	outer membrane lipoprotein A	34	0.0179	0.0109	0.0270	0.0044	O	83%	-
HPSNAG_0784	HPSD74_0959	DNA-binding protein HU-alpha	9	0.0175	0.0384	0	0	C	81%	-
HPSNAG_0889	HPSD74_1113	peptidase M16 inactive domain protein	110	0.0143	0.0128	0.0314	0.0148	P	95%	-
HPSNAG_1000	HPSD74_1053	Tat (twin-arginine translocation) pathway signal sequence domain protein	58	0.0135	0.0070	0.0212	0.0103	P	97%	[70, 72]
HPSNAG_2167	HPSD74_2051	cytochrome b562 family protein	14	0.0134	0.0014	0.0230	0	P	84%	-
HPSNAG_0978	HPSD74_1047	periplasmic solute binding family protein	33	0.0085	0.0023	0.0203	0	P	94%	[70]
HPSNAG_1121	HPSD74_1362	translation elongation factor Tu	43	0.0081	0.0065	0.0140	0.0206	C	91%	-
HPSNAG_0723	HPSD74_0866	penicillin-binding protein activator LpoA	64	0.0076	0.0101	0.0200	0.0151	O	94%	[43]
HPSNAG_0211	HPSD74_0260	maltose-binding periplasmic protein	42	0.0059	0.0018	0.0224	0.0043	P	99%	-
HPSNAG_0140	HPSD74_0186	outer membrane protein P2	39	0.0054	0.0114	0.0132	0.0251	O	75%	[43, 70, 71]
HPSNAG_2042	HPSD74_1917	chaperonin GroL	58	0.0039	0.0106	0.0103	0.0581	C	89%	-
HPSNAG_0493	HPSD74_0594	pyruvate dehydrogenase (acetyl-transferring), homodimeric type	99	0.0020	0.0022	0.0070	0.0206	C	99%	-
HPSNAG_0727	HPSD74_0870	outer membrane autotransporter barrel domain protein (aidA)	91	0.0024	0.0222	0.0012	0.0222	O	68%	[72]

Identified OMV-associated proteins were analyzed for predicted subcellular localization using the pSORTb 3.0 and CELLO2GO programs. Prediction by the pSORTb program was given precedence and results that fell into pSORTb’s Unknown category were then predicted with CELLO2GO. Examination of the mass spectroscopy analysis for the OMV-associated proteins in strain D74 showed that the most abundant proteins from liquid derived samples were predicted to localize to the outer membrane (43%), with cytoplasmic proteins the second most abundant (29%) (Fig 5B). The most abundant D74 plate derived OMV spectral values came from both outer membrane and cytoplasmic proteins (29% for both), with periplasmic proteins the next most abundant (27%). In comparison, the D74 sonicate control consisted primarily of proteins predicted to localize to the cytoplasm (47%). Predicted localization of proteins identified as OMV-associated in strain Nagasaki showed that the most abundant proteins were periplasmic (48% and 43%, liquid and plate), followed by outer membrane proteins (30% and 22%, liquid and plate). Similar to the D74 sonicate control, the most abundant proteins in the Nagasaki sonicate control were predicted to localize to the cytoplasm (43%).

Of the proteins in OMV derived from strain D74, there were outer membrane autotransporters (HPSD74_0166 and HPD74_1658), lipoproteins (HPSD74_1347), a subtilase (HPSD74_1715) and hemagglutinin family proteins (HPSD74_0436 and HPSD74_0814), which were found only in D74, but regardless of growth conditions (liquid and agar) (S1 and S2 Tables). In strain Nagasaki, proteins found in both liquid and plate derived OMV that were missing or reduced in abundance in strain D74 included proteases (HPSNAG_1498 and HPSNAG_2275) and lipoproteins (HPSNAG_1091 and HPSNAG_0421) (S1 and S2 Tables). Examination of the liquid derived OMV-associated proteins between Nagasaki and D74 showed 14 proteins that were more abundant in Nagasaki liquid OMV and 11 proteins that were more abundant in D74 liquid OMV (> 2 fold increase, S1 Table). Similarly, in the plate derived samples, 28 proteins were more abundant in Nagasaki plate OMV and 21 proteins were more abundant in D74 plate OMV (> 2 fold increase, S2 Table). Some of the proteins found to be more abundant in OMV derived from strain Nagasaki were involved in heme utilization (HPSNAG_1011, HPSNAG_2190, HPSNAG_0883, and HPSNAG_0884) as well as an adhesin (HPSNAG_1163). Of the proteins found to be more abundant in purified OMV derived from strain D74, the cytolethal distending toxin protein B (HPSD74_0360) is increased 5–10 fold above the protein level found in Nagasaki OMV and is found in high abundance (NSAF >0.01) under both growth conditions. Additional abundant virulence-associated proteins in OMV derived from strain D74 and Nagasaki included aidA (HPSD74_0870), ompP1 (HPSD74_0351, HPSNAG_0004), ompP2 (HPSD74_0186, HPSNAG_0140) and ompP5 (HPSD74_2216, HPSNAG_2308).

### Host Response to *H*. *parasuis* OMV

**Fig 6:**
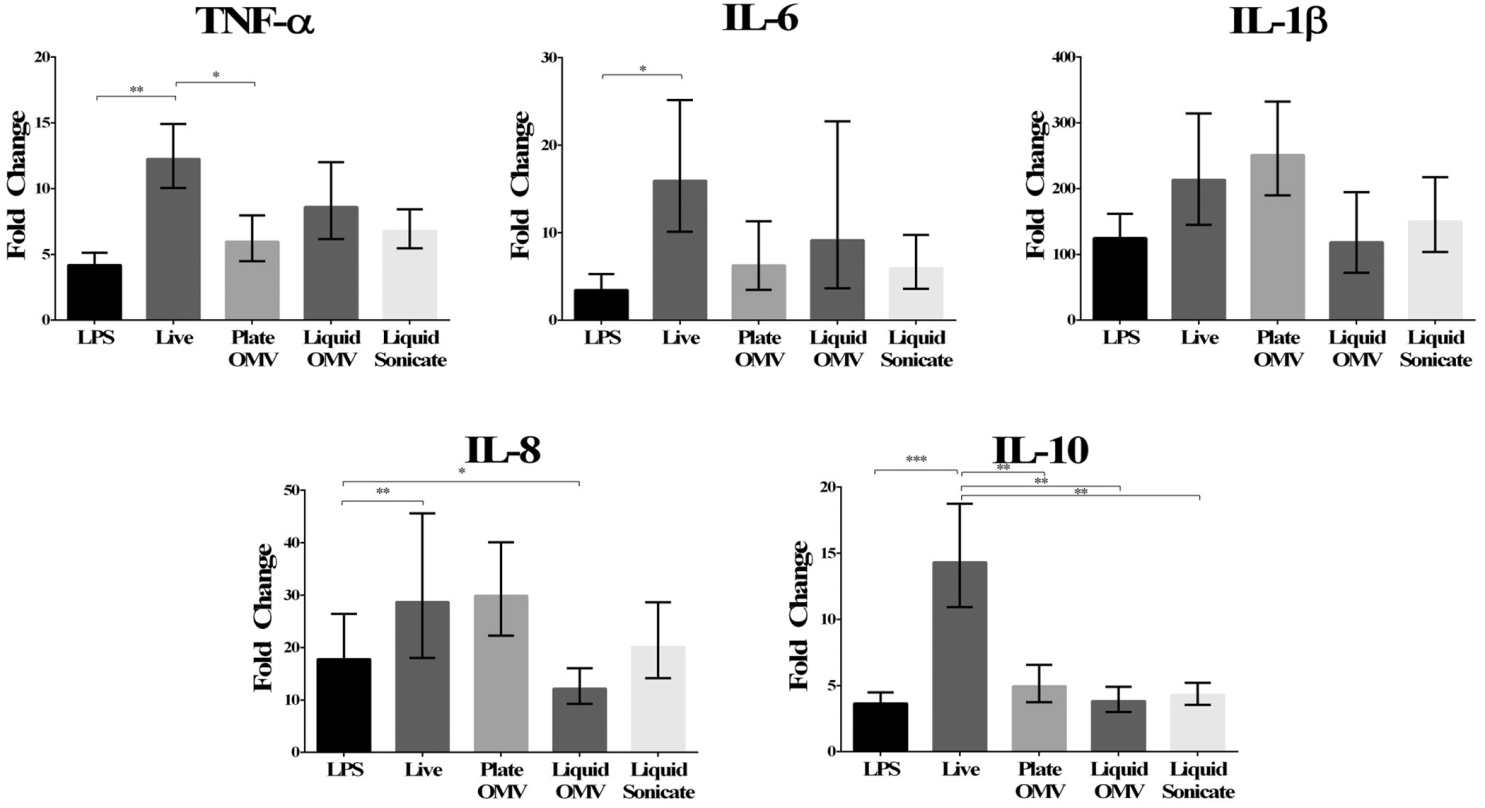
Transcription of cytokine mRNA by porcine alveolar macrophages following stimulation with OMV. Real-time RT-PCR was used to evaluate cytokine mRNA levels in alveolar macrophages 18 h after incubation with *E*. *coli* LPS (10 μg/ml), live *H*. *parasuis* (MOI 2:1), Plate OMV (1 μg), Liquid OMV (1 μg) and Liquid Sonicate (1 μg). Data is expressed as the fold-change in mRNA relative to mock-treated cells. (* = p<0.05, ** = p<0.01, *** = p<0.005).

To evaluate the immunostimulatory capacity of OMV on host cells, we incubated AMac with purified Nagasaki OMV for 18 hours, live *H*. *parasuis*, or *E*. *coli* LPS. RNA was collected from stimulated AMac, and real-time PCR was performed to determine cytokine transcript levels of tumor necrosis factor (TNF)-α, interleukin (IL)-6, IL-1β, IL-8 and IL-10 in response to stimulation relative to non-stimulated cells. Live Nagasaki bacteria induced transcription of all cytokines evaluated above the level of the LPS control, with the exception of IL-1β, where there was no significant difference between any of the treatments (Fig 6). Both liquid- and plate-derived OMV, and sonicate from Nagasaki stimulated the production of transcripts for the proinflammatory cytokines TNF-α, IL-6, IL-1β, and IL-8 at levels that were similar to that of LPS or live bacteria. Only production of the chemokine IL-8 transcript was significantly decreased (p<0.05) in comparison to the LPS control when AMac were stimulated with liquid derived OMV from Nagasaki (Fig 6). Similarly, production of TNF-α transcript was decreased (p<0.05) in comparison to live bacterial stimulation when AMac were stimulated with Nagasaki plate OMV. Transcription of IL-10 was significantly higher in AMac stimulated by live bacteria compared to all other treatments (Fig 6). These results demonstrate that purified OMV and sonicate isolated from strain Nagasaki are able to stimulate cytokine transcription in AMac to similar levels as the live bacteria in most instances (TNF-α plate OMV and IL-10 stimulation being the exceptions). We measured the cytotoxicity of LPS and OMV using a LDH release assay and found that OMV were not significantly cytotoxic to AMac (data not shown).

**Fig 7:**
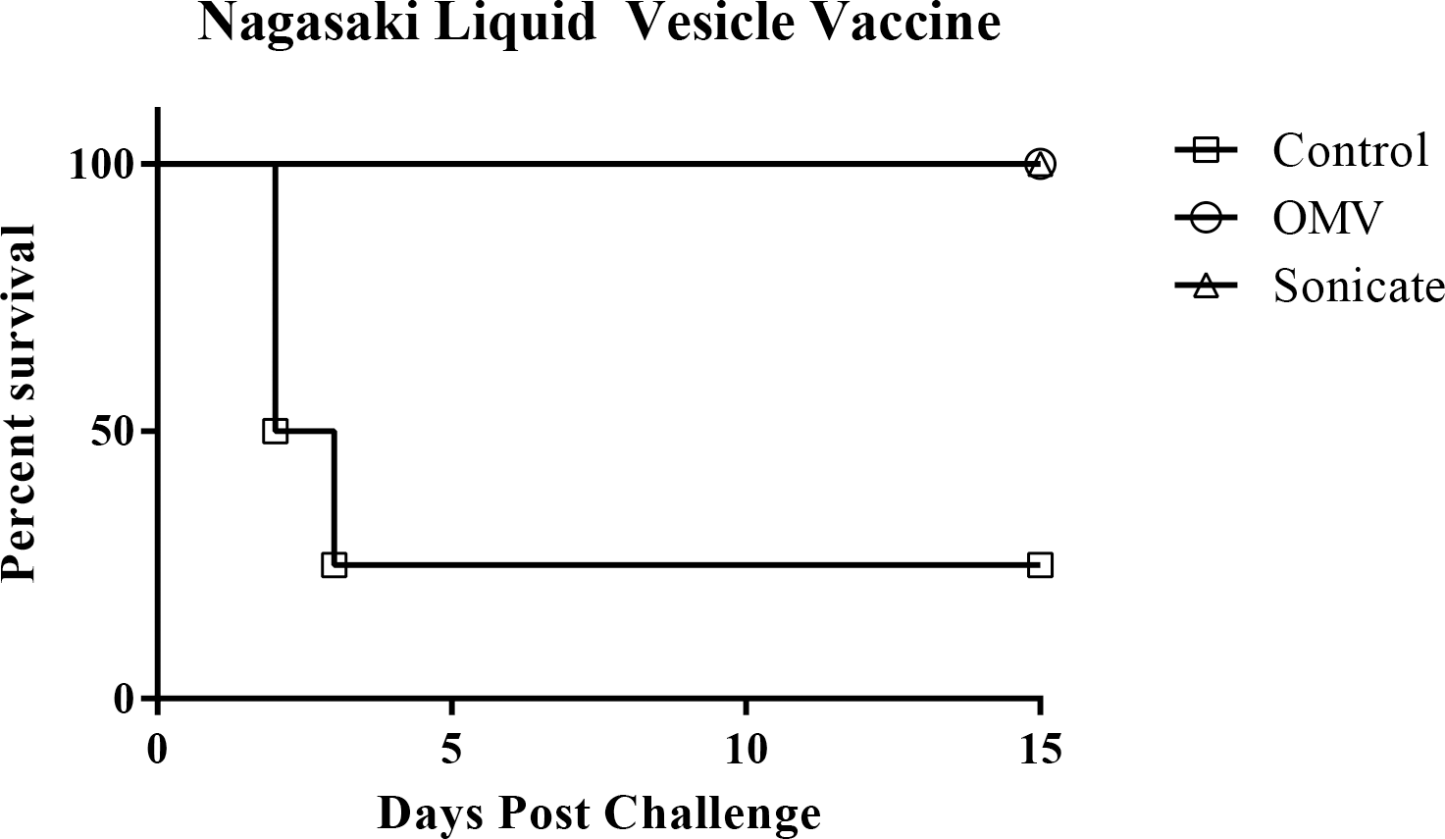
Survival curve Nagasaki outer membrane vesicle and sonicate vaccine. Survival curve of pigs vaccinated with *H*. *parasuis Nagasaki* OMV or sonicate and subsequently challenged intranasally with a lethal dose of Nagasaki.

We vaccinated pigs by intramuscular injection with Nagasaki liquid-derived OMV or liquid-derived sonicate to evaluate the protective capacity of *H. parasuis*-derived OMV. Vaccinated pigs were subsequently challenged with a lethal dose of the Nagasaki strain of *H*. *parasuis* administered by the intranasal route. All vaccinated pigs survived up to 15 days post-intranasal challenge, while 3 of the 4 non-vaccinated control pigs died within 3 days of challenge (Fig 7, p = 0.0028).

**Fig 8:**
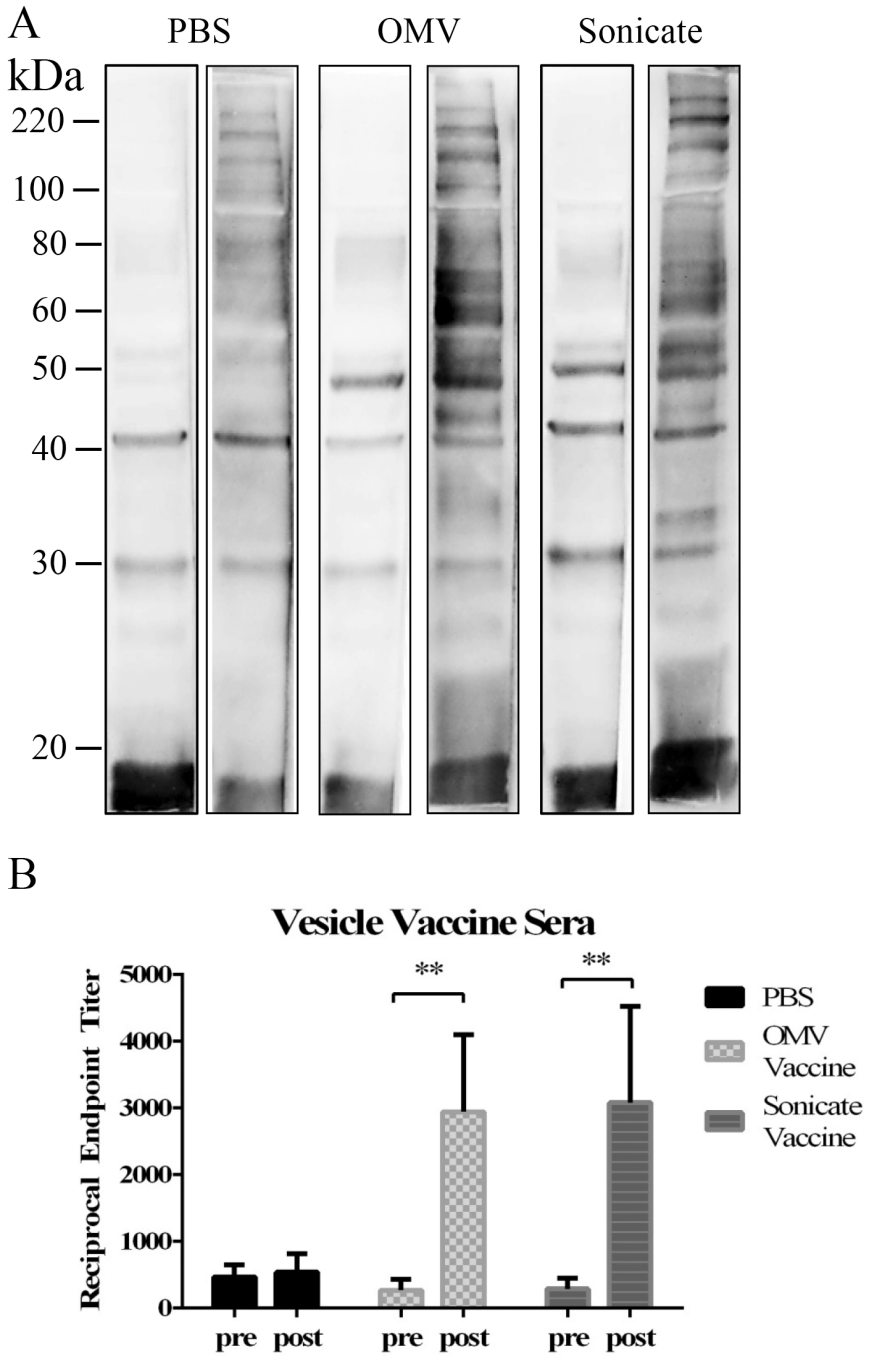
Reactivity of Nagasaki vesicle immunized porcine sera. A. Western blots of Nagasaki whole cell lysate (~10ug) probed with non-vaccinated porcine sera (PBS, left: pre vaccination, right: post PBS vaccination), Nagasaki OMV vaccinated porcine sera (OMV, left: pre-vaccination, right: post OMV vaccination), Nagasaki sonicate vaccinated porcine sera, (Sonicate, left: pre vaccination, right: post sonicate vaccination). B. Endpoint ELISAs performed with sera from pre and post vaccinated pigs (p<0.005).

To evaluate the *H*. *parasuis*-specific antibody response elicited by the Nagasaki OMV and sonicate vaccine, serum was collected from the OMV and sonicate vaccinated pigs before and after vaccination for use in western blots and ELISA. Nagasaki whole cell lysate was separated by SDS-PAGE and Western blots were performed to examine sera reactivity against *H*. *parasuis* proteins (Fig 8A). Sera from the three experimental groups (PBS control, OMV vaccinated, sonicate vaccinated) were pooled by group and vaccine status (pre-vaccine and post-boost). All pig sera collected prior to vaccination had minimal reactivity to *H*. *parasuis* lysate (Fig 8A, left panels). Sera collected post-boost from pigs that received the PBS control vaccine (non-vaccinated) had a slight increase in reactivity to some high molecular weight proteins (> 50 kDa) (Fig 8A, PBS, right panel). In contrast, sera from OMV and sonicate vaccinated pigs reacted to a number of additional *H*. *parasuis* proteins post-vaccination, which was not present when probing with the pre-vaccine sera (Fig 8A, OMV and Sonicate, right panels).

We also performed an endpoint titration ELISA to determine anti-*H*. *parasuis* IgG antibody levels in sera isolated from individual pigs both pre- and post-vaccination. Results showed that vaccination with either OMV or sonicate induced significantly higher anti-*H*. *parasuis* IgG titers (1 LogX greater, OMV p = 0.0028, Sonicate p = 0.0041) when compared to titers for pre-vaccination sera or the PBS control sera (Fig 8B).

## Discussion

This study is the first published report on isolation, characterization and protein identification of OMV from *H*. *parasuis*. A number of complex media and growth conditions were examined to find optimal conditions for growing *H*. *parasuis* for subsequent isolation of OMV. In addition to spherical OMV, tubular structures were observed under different growth conditions in both strains. Similar tubular structures have been reported in a number of other organisms and researchers have demonstrated roles in intercellular communication, attachment to host cells and extracellular electron transport [57–59]. While we have not identified the purpose of these tubular structures in *H*. *parasuis*, the observed increase in production on specific solid growth media does demonstrate a role in interaction with the environment. These data indicate that production of extracellular structures in tested strains of *H*. *parasuis* are influenced by culture media, growth conditions and is strain specific. This also highlights the importance in choice of growth media and cultivation methods when performing both in vivo and in vitro assays.

The OMV isolated and characterized in this study have a number of properties that have previously been observed in structures from other bacterial pathogens, including protein content, protease resistance and the ability to stimulate host cells [60]. Many of the most abundant OMV-associated proteins identified through mass spectrometry have previously been shown to be immunogenic, secreted or virulence factors in *H*. *parasuis* (Table 2). Researchers have isolated OMV from *H*. *influenzae* (also a member of the *Pasteurellaceae* family) and identified associated virulence factors that have homologs in *H*. *parasuis*, which are also OMV-associated [43]. These include proteins involved in heme utilization, as well as the outer membrane porins P2 and P5. We also observed strong reactivity of porcine sera from Nagasaki bacterin vaccinated pigs to purified OMV-associated proteins, confirming that these structures contained numerous antigens. The reactivity of Nagasaki-specific immune sera to the D74 OMV-associated proteins demonstrates that there are immunogenic proteins conserved between the virulent and avirulent strains. We showed that *H*. *parasuis* Nagasaki OMV were able to protect associated proteins from degradation by proteases, similar to OMV studied in other bacteria [60]. Our findings show that naturally produced OMV were better able to protect protein content than a sample consisting of artificially generated vesicles (sonicate). The ability to protect cargo from protease degradation also shows that OMV may function in extracellular secretion in *H*. *parasuis* and confirms that our chosen method is a viable way to obtain OMV from this organism. Our results demonstrated that *H*. *parasuis* OMV are comparable to OMV isolated from other bacterial pathogens and likely contribute to the virulence of this organism.

Proteomic analysis of purified OMV from *H*. *parasuis* identified a number of differences in protein content between strains and growth conditions. OMV isolated from bacteria grown in liquid media had 101 unique associated proteins, while plate grown strains had 284 unique associated proteins. The majority of the OMV-associated protein spectra (>70% of NSAF values) were found in both strains when the samples were isolated from bacteria propagated under the same growth conditions. A number of proteins that were found in OMV from both strains were predicted to localize to the cytoplasm, including chaperonin GroL (HPSD74_1917, HPSNAG_2042), DNA-binding protein HU-alpha (HPSD74_0959, HPSNAG_0784), translation elongation factor G (HPSNAG_1930) and translation elongation factor Tu (HPSD74_1362, HPSNAG_1121). The number of cytoplasmic proteins associated with OMV may be the result of bacterial lysis and association of proteins with the newly formed vesicles, or may be a type of vesicle that has been recently described, an outer-inner membrane vesicle (O-IMV) which are enriched in cytoplasmic proteins [61]. It is worth noting that predicted cytoplasmic proteins in the sonicate control samples were more abundant (47% and 43%) than in any of the naturally produced OMV samples, which indicates that the number of proteins associated with OMV were not solely due to bacterial lysis. Additionally, a number of bacterial OMV have been examined in other organisms and some researchers have reported a high percentage (> 30%) of cytoplasmic proteins associated with these structures [62, 63]. Since OMV isolated from both strains were not significantly cytotoxic to porcine AMac (data not shown), it remains to be discovered what benefit, if any, packaging these proteins into OMV provides the bacteria. The high abundance of some proteins in OMV would be the result of their abundance in the outer membrane of *H*. *parasuis*–thus, the proteins are included in vesicles as they are formed and inclusion is unrelated to pathogenesis.

A number of biological interactions between bacterial OMV and host cells have been examined in other organisms, including the ability of these structures to manipulate host immunity and facilitate host cell death [32, 60]. OMV from D74 and Nagasaki were not significantly cytotoxic to AMac, however, they were capable of stimulating cytokine transcription to similar levels as live bacteria. Thus, OMV in *H*. *parasuis* function in immune modulation in the host, a role that has been reported in other bacterial pathogens [43]. OMV derived from liquid grown Nagasaki were able to stimulate production of the proinflammatory cytokines TNF-α, IL-6 and IL-1β to levels that were not significantly different from live bacteria. Plate derived OMV were able to stimulate IL-6, IL-1β and IL-8 to levels comparable to the live bacteria. These results are in agreement with findings by other researchers in which OMV produced by *H*. *influenzae*, *Helicobacter*, *Klebsiella* and *Pseudomonas* were shown to stimulate production of the cytokines TNF-α, IL-1β, IL-6 and IL-8 [43, 64–67]. Interestingly, production of the anti-inflammatory cytokine IL-10 by AMac was not significantly increased in comparison to live bacteria for any of the Nagasaki vesicles tested (liquid, plate or sonicate). This finding is in contrast to results previously seen by other researchers stimulating host dendritic cells with OMV isolated from *Neisseria meningitidis*, in which significant production of IL-10 was observed [68]. This may indicate that cell types are differentially effected by OMV (alveolar macrophages versus dendritic cells), that the observed level of stimulation is the maximum for the concentration of OMV used, or alternatively, that the live bacteria are somehow able to stimulate an IL-10 response [69], but OMV does not (lowering the immune response). Further research with *H*. *parasuis* OMV and other cell types may help to determine what role the OMV may be playing in immune modulation of the host.

Vaccination with both OMV derived from Nagasaki bacteria grown in liquid culture and liquid grown Nagasaki sonicate protected pigs from lethal challenge with strain Nagasaki. It’s important to note that the OMV and sonicate were delivered without the addition of any adjuvant, unlike the commercial OMV vaccine against *Neisseria meningitidis*. Liquid-derived OMV from strain Nagasaki also contained the fewest proteins of any of the OMV or sonicate preparations but was still protective, narrowing the list of potential candidates for use in a subunit vaccine. Our results showed that the observed protection was associated with an antibody response to specific OMV-associated proteins and confirmed that there was a significant increase in peripheral *H*. *parasuis*-specific antibody in vaccinated pigs. Importantly, in our ELISA experiment, pre-challenge sera from the single surviving control pig did not exhibit increased reactivity above that of the other control pigs, evidence that survival was not due to prior exposure to *Haemophilus*. The surviving control pig was derived from a separate litter than the other vaccinated animals in this experiment suggesting individual genetic background may be important in resistance to infection with *H*. *parasuis*. These results demonstrate that OMV and sonicate derived from a pathogenic strain of *H*. *parasuis* were capable of eliciting a protective immune response. This also highlights the natural immunogenic properties of OMV, as these structures were given to the host without the use of an adjuvant.

The use of OMV as vaccine components has been researched by a number of groups and a commercial vaccine against *N*. *meningitides* has been used to successfully protect against disease [39]. While OMV production and host response to these structures have been studied for nearly 30 years in the related *H*. *influenzae*, no research has been performed on these subjects in *H*. *parasuis*. Researchers have examined the use of nontypeable *H*. *influenzae* OMV as a potential vaccine, discovered that OMV are able to induce cross-protective immunity in mice and are capable of diverting the host humoral response via a novel virulence mechanism [35]. Currently the preferred method of protecting swine involves vaccination with bacterin based vaccines; however, the efficacy of these are limited and cross-protection against additional strains of *H*. *parasuis* is unpredictable [11]. It may be possible to create a multivalent vaccine using OMV (or sonicate) from numerous strains of *H*. *parasuis* that can provide protection against multiple strains. Examination of OMV from additional strains of *H*. *parasuis*, as well as their effect on additional host cell types, will give more insight into the virulence factors utilized by this organism to cause disease in swine, as well as provide useful targets for inclusion into a vaccine.

## Supporting Information

S1 Fig Growth curves. Representative growth curve for *Haemophilus parasuis* Nagasaki and D74 grown in BHI media.
(TIF)

S2 Fig Transmission electron microscopy of *H*. *parasuis* grown in different media and growth conditions. A. D74 BHI liquid, B. D74 BHI plate, C. D74 Casmans plate, D. D74 TSA plate, E. Nagasaki BHI liquid, F. Nagasaki BHI plate, G. Nagasaki Casmans plate, H. Nagasaki TSA plate, scale bar = 1 micron.
(TIF)

S1 Table D74 and Nagasaki Liquid OMV-associated proteins. (DOCX)

S2 Table D74 and Nagasaki Plate OMV-associated proteins. (DOCX)
