# Supplementary figures and images for: Characterization and Vaccine Potential of Outer Membrane Vesicles Produced by Haemophilus parasuis
Source: PLoS One. 2016 Mar 1;11(3):e0149132. doi: 10.1371/journal.pone.0149132 (PMC4773134; doi:10.1371/journal.pone.0149132)

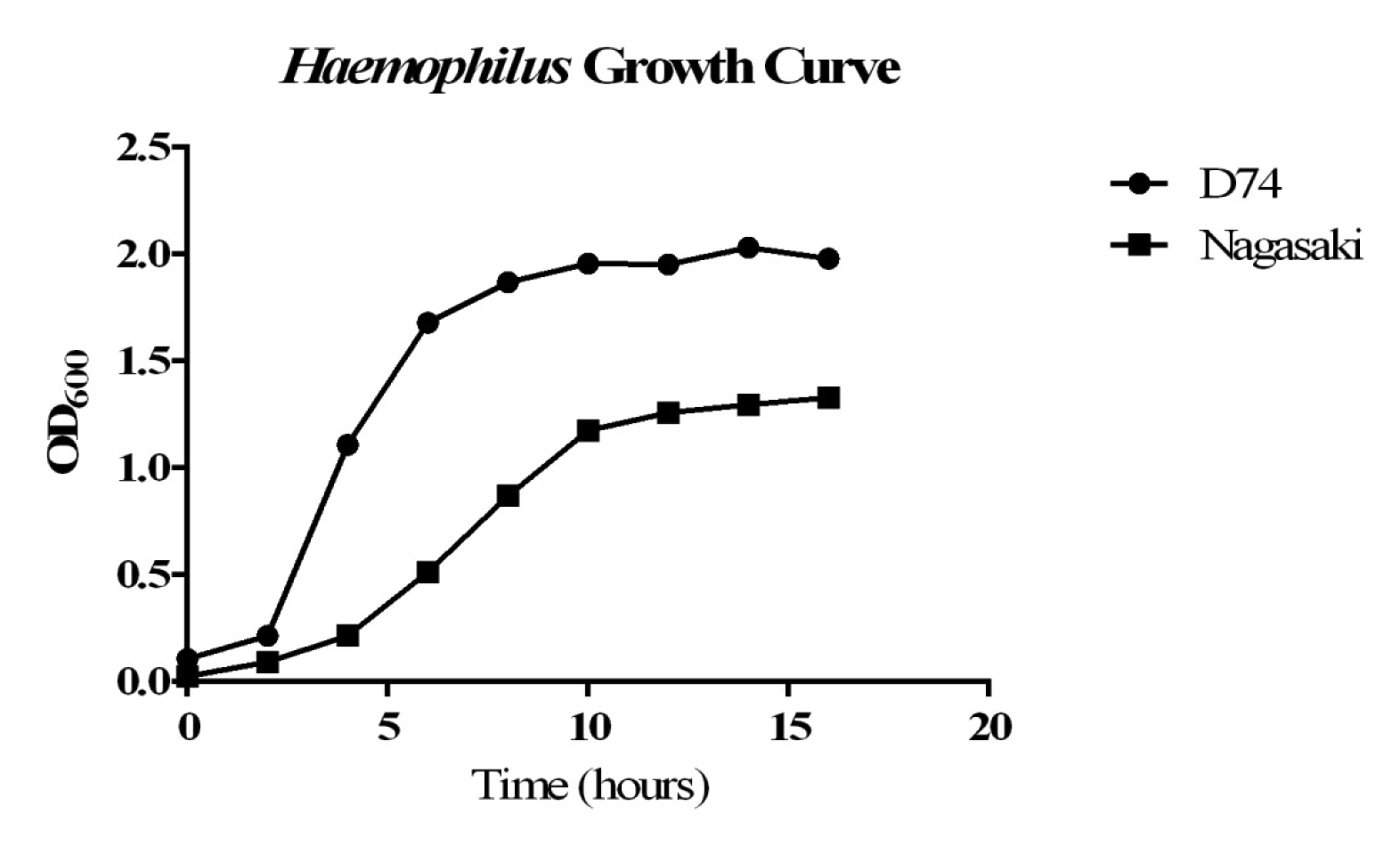

Supplement: S1 Fig — Representative growth curve for Haemophilus parasuis Nagasaki and D74 grown in BHI media. (TIF) [file pone.0149132.s001.tif]

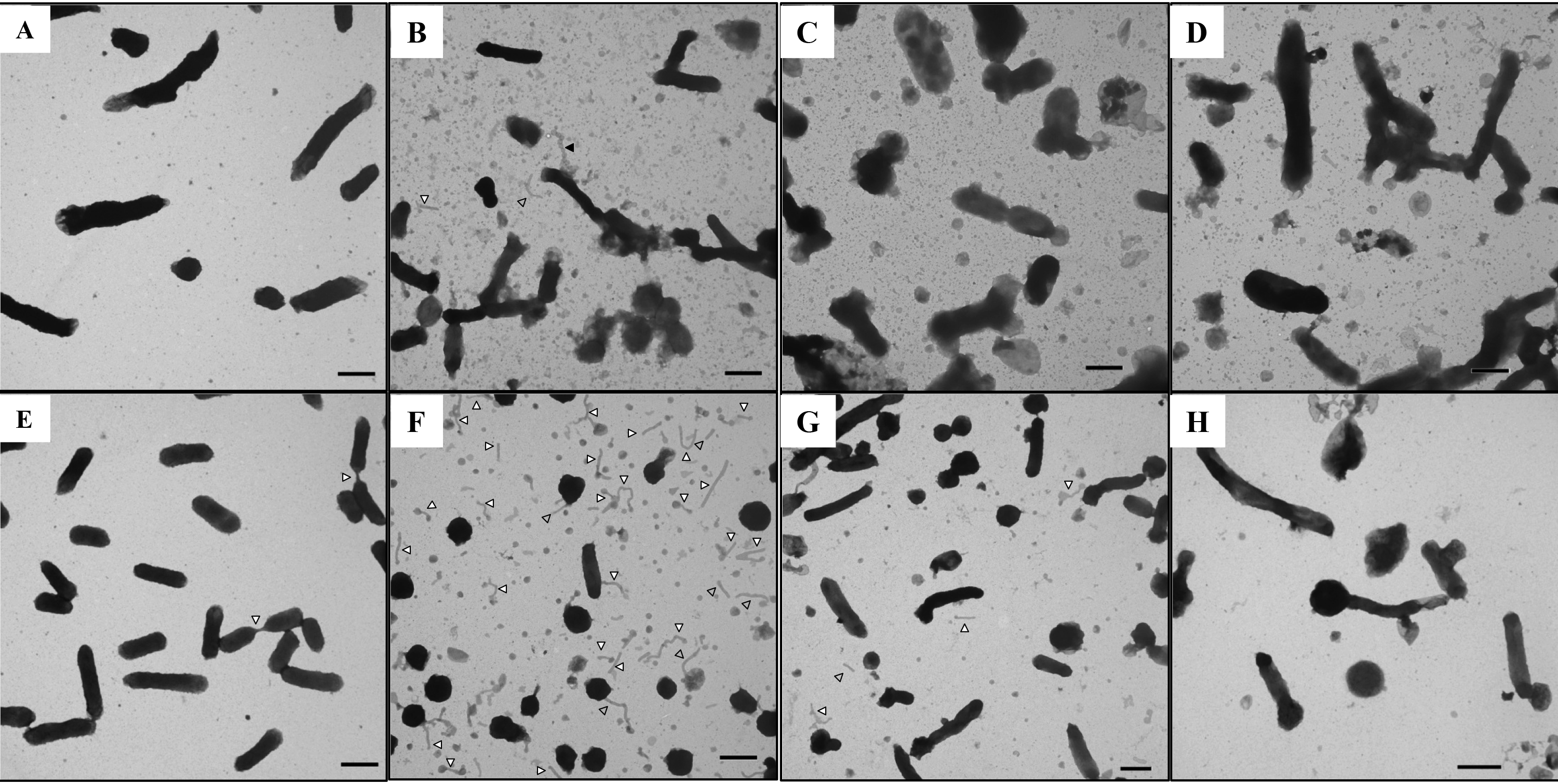

Supplement: S2 Fig — A. D74 BHI liquid, B. D74 BHI plate, C. D74 Casmans plate, D. D74 TSA plate, E. Nagasaki BHI liquid, F. Nagasaki BHI plate, G. Nagasaki Casmans plate, H. Nagasaki TSA plate, scale bar = 1 micron. (TIF) [file pone.0149132.s002.tif]
