# Supplementary material for: Characterization and Vaccine Potential of Outer Membrane Vesicles Produced by Haemophilus parasuis
Source: PLoS One. 2016 Mar 1;11(3):e0149132. doi: 10.1371/journal.pone.0149132 (PMC4773134; doi:10.1371/journal.pone.0149132)
Supplement: S1 Table — (DOCX) [file pone.0149132.s003.docx]

**Supplementary Table S2: D74 and Nagasaki Liquid OMV-associated proteins.**

| **Nagasaki Locus** | **D74 Locus** | **Protein Description** | **Nagasaki**  **NSAF** | **D74**  **NSAF** | **Percent Similarity** | **Fold Change** |
| --- | --- | --- | --- | --- | --- | --- |
| HPSNAG_0889 | HPSD74_1113 | peptidase M16 inactive domain protein | 0.0314 | 0.0148 | 0.95 | 2.12 |
| HPSNAG_0042 | HPSD74_0020 | peptidoglycan-associated lipoprotein | 0.0288 | 0.0233 | 0.89 | 1.24 |
| HPSNAG_1330 | HPSD74_1964 | outer membrane lipoprotein A | 0.0270 | 0.0044 | 0.83 | 6.09 |
| HPSNAG_0730 | HPSD74_0873 | superoxide dismutase | 0.0259 | 0.0000 | 0.88 | 0.00 |
| HPSNAG_0439 | HPSD74_0534 | D-galactose-binding periplasmic protein | 0.0244 | 0.0061 | 0.96 | 4.01 |
| HPSNAG_2167 | HPSD74_2051 | cytochrome b562 family protein | 0.0230 | 0.0000 | 0.84 | 0.00 |
| HPSNAG_0211 | HPSD74_0260 | maltose-binding periplasmic protein | 0.0224 | 0.0043 | 0.99 | 5.21 |
| HPSNAG_2308 | HPSD74_2216 | outer membrane protein P5 | 0.0218 | 0.0345 | 0.81 | -1.58 |
| HPSNAG_1000 | HPSD74_1053 | Tat (twin-arginine translocation) pathway signal sequence domain protein | 0.0212 | 0.0103 | 0.97 | 2.06 |
| HPSNAG_0978 | HPSD74_1047 | periplasmic solute binding family protein | 0.0203 | 0.0000 | 0.94 | 0.00 |
| HPSNAG_0723 | HPSD74_0866 | penicillin-binding protein activator LpoA | 0.0200 | 0.0151 | 0.94 | 1.33 |
| HPSNAG_1757 | HPSD74_1476 | holliday junction resolvase | 0.0190 | 0.0174 | 1.00 | 1.09 |
| HPSNAG_0049 | HPSD74_0027 | bacterial extracellular solute-binding family protein | 0.0188 | 0.0115 | 0.99 | 1.63 |
| HPSNAG_1687 | HPSD74_1634 | FKBP-type peptidyl-prolyl cis-trans isomerase fkpA | 0.0167 | 0.0046 | 0.98 | 3.61 |
| HPSNAG_1498 | none | protease Do family protein | 0.0156 | 0.0000 | - | 0.00 |
| HPSNAG_1011 | HPSD74_1065 | iron binding protein FbpA | 0.0148 | 0.0118 | 0.92 | 1.26 |
| HPSNAG_0443 | HPSD74_0538 | putative D-methionine-binding lipoprotein metQ | 0.0141 | 0.0149 | 0.82 | -1.06 |
| HPSNAG_1121 | HPSD74_1362 | translation elongation factor Tu | 0.0140 | 0.0206 | 0.91 | -1.47 |
| HPSNAG_1163 | HPSD74_1404 | outer membrane family protein | 0.0140 | 0.0033 | 0.93 | 4.25 |
| HPSNAG_1464 | HPSD74_1577 | domain amino terminal to FKBP-type peptidyl-prolyl isomerase family protein | 0.0136 | 0.0157 | 0.98 | -1.15 |
| HPSNAG_0041 | HPSD74_0019 | Tol-Pal system beta propeller repeat protein TolB | 0.0136 | 0.0032 | 0.91 | 4.20 |
| HPSNAG_1095 | HPSD74_1165 | heme-binding protein A | 0.0135 | 0.0124 | 0.96 | 1.09 |
| HPSNAG_1627 | HPSD74_1712 | D-ribose-binding periplasmic protein | 0.0133 | 0.0000 | 0.90 | 0.00 |
| HPSNAG_0140 | HPSD74_0186 | outer membrane protein P2 | 0.0132 | 0.0251 | 0.75 | -1.90 |
| HPSNAG_0142 | HPSD74_0188 | bacterial extracellular solute-binding family protein | 0.0128 | 0.0068 | 0.92 | 1.88 |
| HPSNAG_2232 | HPSD74_2125 | transferrin-binding protein 2 | 0.0128 | 0.0130 | 0.93 | -1.02 |
| HPSNAG_0479 | HPSD74_0580 | protein UshA | 0.0119 | 0.0084 | 0.93 | 1.43 |
| HPSNAG_0720 | HPSD74_0863 | BON domain protein | 0.0114 | 0.0139 | 0.88 | -1.22 |
| HPSNAG_0421 | none | putative lipoprotein | 0.0113 | 0.0000 | - | 0.00 |
| HPSNAG_0682 | HPSD74_0099 | hypothetical protein | 0.0113 | 0.0076 | 0.98 | 1.48 |
| HPSNAG_1162 | HPSD74_1403 | outer membrane assembly complex, YaeT protein | 0.0113 | 0.0085 | 0.99 | 1.33 |
| HPSNAG_1930 | HPSD74_2170 | translation elongation factor G | 0.0111 | 0.0021 | 0.95 | 5.26 |
| HPSNAG_0617 | HPSD74_0784 | subtilase family protein | 0.0109 | 0.0085 | 0.96 | 1.28 |
| HPSNAG_1476 | none | BNR/Asp-box repeat family protein | 0.0106 | 0.0000 | - | 0.00 |
| HPSNAG_1769 | HPSD74_1488 | hypothetical protein | 0.0104 | 0.0117 | 0.92 | -1.13 |
| HPSNAG_2042 | HPSD74_1917 | chaperonin GroL | 0.0103 | 0.0581 | 0.89 | -5.66 |
| HPSNAG_0859 | HPSD74_1459 | glucose-specific phosphotransferase enzyme IIA component | 0.0099 | 0.0055 | 0.99 | 1.79 |
| HPSNAG_0876 | HPSD74_1100 | periplasmic oligopeptide-binding protein | 0.0098 | 0.0000 | 0.88 | 0.00 |
| HPSNAG_2233 | HPSD74_2126 | transferrin-binding protein 1 | 0.0094 | 0.0107 | 0.84 | -1.14 |
| HPSNAG_0243 | none | smpA / OmlA family protein | 0.0092 | 0.0000 | - | 0.00 |
| HPSNAG_2279 | HPSD74_2190 | putative phospholipid-binding lipoprotein mlaA | 0.0084 | 0.0077 | 0.98 | 1.10 |
| HPSNAG_0910 | HPSD74_1714 | autotransporter beta-domain protein | 0.0078 | 0.0042 | 0.89 | 1.87 |
| HPSNAG_0204 | HPSD74_0253 | lipoprotein copper homeostasis and adhesion, NlpE | 0.0078 | 0.0118 | 0.96 | -1.52 |
| HPSNAG_0676 | HPSD74_0105 | small protein A | 0.0076 | 0.0178 | 0.90 | -2.34 |
| HPSNAG_0131 | HPSD74_0176 | formate acetyltransferase | 0.0070 | 0.0026 | 0.98 | 2.69 |
| HPSNAG_0493 | HPSD74_0594 | pyruvate dehydrogenase (acetyl-transferring), homodimeric type | 0.0070 | 0.0206 | 0.99 | -2.95 |
| HPSNAG_1662 | HPSD74_1608 | putative lipoprotein | 0.0067 | 0.0064 | 0.98 | 1.04 |
| HPSNAG_1845 | HPSD74_0695 | lipoprotein nlpD | 0.0064 | 0.0055 | 0.84 | 1.17 |
| HPSNAG_0644 | HPSD74_0845 | hypothetical protein | 0.0063 | 0.0026 | 0.91 | 2.43 |
| HPSNAG_0158 | HPSD74_0204 | disulfide interchange protein DsbC | 0.0062 | 0.0053 | 0.99 | 1.17 |
| HPSNAG_0884 | HPSD74_1108 | hemX family protein | 0.0062 | 0.0013 | 0.93 | 4.75 |
| HPSNAG_2024 | HPSD74_1910 | ribose-phosphate pyrophosphokinase | 0.0058 | 0.0066 | 1.00 | -1.14 |
| HPSNAG_0492 | none | dihydrolipoyllysine-residue acetyltransferase | 0.0056 | 0.0114 | 0.94 | -2.04 |
| HPSNAG_1298 | HPSD74_1320 | L-cystine-binding protein tcyA | 0.0055 | 0.0125 | 0.98 | -2.26 |
| HPSNAG_1773 | HPSD74_1492 | bamD | 0.0055 | 0.0074 | 0.93 | -1.36 |
| HPSNAG_0038 | HPSD74_0016 | protein TolQ | 0.0052 | 0.0000 | 0.85 | 0.00 |
| HPSNAG_1877 | HPSD74_2005 | TRAP transporter solute receptor, TAXI family protein | 0.0051 | 0.0033 | 0.94 | 1.55 |
| HPSNAG_1625 | none | glutamine synthetase, type I | 0.0050 | 0.0000 | - | 0.00 |
| HPSNAG_2190 | HPSD74_2080 | heme/hemopexin-binding protein | 0.0047 | 0.0009 | 0.90 | 5.23 |
| HPSNAG_1355 | HPSD74_1988 | hemoglobin-binding protease hbp autotransporter | 0.0047 | 0.0000 | 0.96 | 0.00 |
| HPSNAG_0034 | HPSD74_0012 | cytochrome bd-I terminal oxidase subunit I | 0.0043 | 0.0047 | 0.94 | -1.09 |
| HPSNAG_1590 | HPSD74_1787 | disulfide interchange protein DsbA | 0.0042 | 0.0049 | 1.00 | -1.15 |
| HPSNAG_2169 | HPSD74_2053 | adenylosuccinate synthase | 0.0042 | 0.0044 | 0.98 | -1.04 |
| HPSNAG_0060 | HPSD74_0037 | heme-binding protein A | 0.0041 | 0.0030 | 0.95 | 1.36 |
| HPSNAG_0239 | HPSD74_0361 | cytolethal distending toxin subunit A | 0.0037 | 0.0063 | 0.90 | -1.70 |
| HPSNAG_0491 | HPSD74_0592 | dihydrolipoyl dehydrogenase | 0.0034 | 0.0197 | 0.92 | -5.73 |
| HPSNAG_2188 | HPSD74_2078 | heme/hemopexin utilization protein C | 0.0033 | 0.0061 | 0.95 | -1.83 |
| HPSNAG_1763 | HPSD74_1482 | bacterial extracellular solute-binding s, 5 Middle family protein | 0.0030 | 0.0014 | 0.94 | 2.17 |
| HPSNAG_0920 | HPSD74_1263 | ABC transporter arginine-binding protein | 0.0028 | 0.0072 | 0.95 | -2.63 |
| HPSNAG_2293 | HPSD74_2203 | penicillin-binding protein 1A | 0.0025 | 0.0000 | 0.97 | 0.00 |
| HPSNAG_0238 | HPSD74_0282 | cytolethal distending toxin protein B | 0.0024 | 0.0000 | 1.00 | 0.00 |
| HPSNAG_0662 | HPSD74_0119 | phenylalanine--tRNA ligase, alpha subunit | 0.0021 | 0.0154 | 1.00 | -7.43 |
| HPSNAG_2140 | HPSD74_2247 | S-adenosyl-l-methionine hydroxide adenosyltransferase family protein | 0.0020 | 0.0036 | 0.99 | -1.76 |
| HPSNAG_0296 | HPSD74_0360 | cytolethal distending toxin protein B | 0.0019 | 0.0105 | 0.93 | -5.45 |
| HPSNAG_0960 | HPSD74_1029 | ubiquinone oxidoreductase, Na(+)-translocating, A subunit | 0.0019 | 0.0041 | 0.91 | -2.20 |
| HPSNAG_0727 | HPSD74_0870 | outer membrane autotransporter barrel domain protein (aidA) | 0.0012 | 0.0222 | 0.68 | -17.87 |
| HPSNAG_1938 | none | valine--tRNA ligase | 0.0012 | 0.0000 | - | 0.00 |
| HPSNAG_2275 | HPSD74_2186 | tail-specific protease | 0.0010 | 0.0000 | 0.98 | 0.00 |
| none | HPSD74_1347 | outer membrane lipoprotein pcp | 0.0000 | 0.0155 | - | 0.00 |
| none | HPSD74_1169 | hypothetical protein | 0.0000 | 0.0127 | - | 0.00 |
| HPSNAG_1020 | HPSD74_1074 | 6, 7-dimethyl-8-ribityllumazine synthase | 0.0000 | 0.0126 | 0.99 | 0.00 |
| none | HPSD74_1658 | outer membrane autotransporter barrel domain protein | 0.0000 | 0.0113 | - | 0.00 |
| HPSNAG_0663 | HPSD74_0118 | phenylalanyl-tRNA synthetase, beta subunit | 0.0000 | 0.0104 | 0.96 | 0.00 |
| none | HPSD74_0280 | hypothetical protein | 0.0000 | 0.0098 | - | 0.00 |
| none | HPSD74_1916 | tonB-dependent Receptor Plug domain protein | 0.0000 | 0.0098 | - | 0.00 |
| HPSNAG_0122 | HPSD74_0166 | outer membrane autotransporter barrel domain protein | 0.0000 | 0.0089 | 0.79 | 0.00 |
| HPSNAG_1084 | HPSD74_0351 | outer membrane protein P1 | 0.0000 | 0.0088 | 0.89 | 0.00 |
| HPSNAG_0725 | HPSD74_0868 | DNA protection during starvation protein | 0.0000 | 0.0071 | - | 0.00 |
| none | HPSD74_1715 | subtilase family protein | 0.0000 | 0.0066 | - | 0.00 |
| HPSNAG_2342 | HPSD74_0874 | transposase, Mutator family protein | 0.0000 | 0.0063 | 1.00 | 0.00 |
| none | HPSD74_1529 | hypothetical protein | 0.0000 | 0.0051 | - | 0.00 |
| none | HPSD74_0044 | tonB-dependent siderophore receptor family protein | 0.0000 | 0.0050 | - | 0.00 |
| none | HPSD74_0696 | lysM domain protein | 0.0000 | 0.0048 | - | 0.00 |
| none | HPSD74_2044 | putative tonB-dependent receptor | 0.0000 | 0.0046 | - | 0.00 |
| none | HPSD74_1526 | hypothetical protein | 0.0000 | 0.0044 | - | 0.00 |
| HPSNAG_0623 | HPSD74_0792 | dihydrolipoyllysine-residue succinyltransferase, E2 component of oxoglutarate dehydrogenase | 0.0000 | 0.0037 | 0.99 | 0.00 |
| HPSNAG_2310 | HPSD74_2219 | glyceraldehyde-3-phosphate dehydrogenase, type I | 0.0000 | 0.0033 | 0.91 | 0.00 |
| none | HPSD74_0579 | tonB-dependent siderophore receptor family protein | 0.0000 | 0.0028 | - | 0.00 |
| none | HPSD74_0814 | filamentous hemagglutinin family N-terminal domain protein | 0.0000 | 0.0021 | - | 0.00 |
| none | HPSD74_0436 | filamentous hemagglutinin family N-terminal domain protein | 0.0000 | 0.0012 | - | 0.00 |
