# Supplementary material for: Characterization and Vaccine Potential of Outer Membrane Vesicles Produced by Haemophilus parasuis
Source: PLoS One. 2016 Mar 1;11(3):e0149132. doi: 10.1371/journal.pone.0149132 (PMC4773134; doi:10.1371/journal.pone.0149132)
Supplement: S2 Table — (DOCX) [file pone.0149132.s004.docx]

**Supplementary Table S2: D74 and Nagasaki Plate OMV-associated proteins.**

| **Nagasaki Locus** | **D74**  **Locus** | **Protein Description** | **Nagasaki NSAF** | **D74**  **NSAF** | **Percent Similarity** | **Fold Change** |
| --- | --- | --- | --- | --- | --- | --- |
| HPSNAG_0042 | HPSD74_0020 | peptidoglycan-associated lipoprotein | 0.0297 | 0.0116 | 0.89 | 2.56 |
| HPSNAG_2308 | HPSD74_2216 | outer membrane protein P5 | 0.0276 | 0.0191 | 0.81 | 1.44 |
| HPSNAG_1095 | HPSD74_1165 | heme-binding protein A | 0.0270 | 0.0162 | 0.96 | 1.66 |
| HPSNAG_1298 | HPSD74_1320 | L-cysteine-binding protein tcyA | 0.0265 | 0.0239 | 0.98 | 1.11 |
| HPSNAG_0439 | HPSD74_0534 | D-galactose-binding periplasmic protein | 0.0230 | 0.0076 | 0.96 | 3.01 |
| HPSNAG_0730 | HPSD74_0873 | superoxide dismutase | 0.0192 | 0.0000 | 0.88 | 0 |
| HPSNAG_1330 | HPSD74_1964 | outer membrane lipoprotein A | 0.0179 | 0.0109 | 0.83 | 1.65 |
| HPSNAG_1476 | none | BNR/Asp-box repeat family protein | 0.0179 | 0.0000 | 0 | 0 |
| HPSNAG_0784 | HPSD74_0959 | DNA-binding protein HU-alpha | 0.0175 | 0.0384 | 0.81 | -2.19 |
| HPSNAG_0049 | HPSD74_0027 | bacterial extracellular solute-binding family protein | 0.0161 | 0.0058 | 0.99 | 2.76 |
| HPSNAG_0889 | HPSD74_1113 | peptidase M16 inactive domain protein | 0.0143 | 0.0128 | 0.95 | 1.12 |
| HPSNAG_1000 | HPSD74_1053 | Tat (twin-arginine translocation) pathway signal sequence domain protein | 0.0135 | 0.0070 | 0.97 | 1.93 |
| HPSNAG_2167 | HPSD74_2051 | cytochrome b562 family protein | 0.0134 | 0.0014 | 0.84 | 9.26 |
| HPSNAG_1769 | HPSD74_1488 | hypothetical protein | 0.0127 | 0.0085 | 0.92 | 1.50 |
| HPSNAG_1011 | HPSD74_1065 | iron binding protein FbpA | 0.0124 | 0.0044 | 0.92 | 2.83 |
| HPSNAG_0243 | none | smpA / OmlA family protein | 0.0114 | 0.0000 | 0 | 0 |
| HPSNAG_0060 | HPSD74_0037 | heme-binding protein A | 0.0107 | 0.0073 | 0.95 | 1.47 |
| HPSNAG_1627 | HPSD74_1712 | D-ribose-binding periplasmic protein | 0.0107 | 0.0038 | 0.9 | 2.80 |
| HPSNAG_0142 | HPSD74_0188 | bacterial extracellular solute-binding family protein | 0.0099 | 0.0076 | 0.92 | 1.31 |
| HPSNAG_1163 | HPSD74_1404 | outer membrane family protein | 0.0099 | 0.0044 | 0.93 | 2.24 |
| HPSNAG_0910 | HPSD74_1714 | autotransporter beta-domain protein | 0.0092 | 0.0053 | 0.89 | 1.72 |
| HPSNAG_1498 | none | protease Do family protein | 0.0089 | 0.0000 | 0 | 0 |
| HPSNAG_1687 | HPSD74_1634 | FKBP-type peptidyl-prolyl cis-trans isomerase fkpA | 0.0088 | 0.0048 | 0.98 | 1.82 |
| HPSNAG_0978 | HPSD74_1047 | periplasmic solute binding family protein | 0.0085 | 0.0023 | 0.94 | 3.61 |
| HPSNAG_1121 | HPSD74_1362 | translation elongation factor Tu | 0.0081 | 0.0065 | 0.91 | 1.26 |
| HPSNAG_0720 | HPSD74_0863 | BON domain protein | 0.0081 | 0.0060 | 0.88 | 1.35 |
| HPSNAG_1464 | HPSD74_1577 | domain amino terminal to FKBP-type peptidyl-prolyl isomerase family protein | 0.0081 | 0.0129 | 0.98 | -1.59 |
| HPSNAG_1162 | HPSD74_1403 | outer membrane assembly complex, YaeT protein | 0.0080 | 0.0064 | 0.99 | 1.25 |
| HPSNAG_0204 | HPSD74_0253 | lipoprotein copper homeostasis and adhesion, NlpE | 0.0079 | 0.0076 | 0.96 | 1.03 |
| HPSNAG_0682 | HPSD74_0099 | hypothetical protein | 0.0079 | 0.0069 | 0.98 | 1.14 |
| HPSNAG_0479 | HPSD74_0580 | protein UshA | 0.0078 | 0.0090 | 0.93 | -1.15 |
| HPSNAG_1637 | none | ATP synthase F0, B subunit | 0.0077 | 0.0000 | 0 | 0 |
| HPSNAG_1757 | HPSD74_1476 | holliday junction resolvase | 0.0077 | 0.0087 | 1 | -1.13 |
| HPSNAG_0723 | HPSD74_0866 | penicillin-binding protein activator LpoA | 0.0076 | 0.0101 | 0.94 | -1.34 |
| HPSNAG_0676 | HPSD74_0105 | small protein A | 0.0074 | 0.0094 | 0.9 | -1.28 |
| HPSNAG_0443 | HPSD74_0538 | putative D-methionine-binding lipoprotein metQ | 0.0072 | 0.0057 | 0.82 | 1.27 |
| HPSNAG_0716 | HPSD74_0859 | putative phospholipid-binding protein mlaC | 0.0071 | 0.0030 | 0.99 | 2.37 |
| HPSNAG_0870 | HPSD74_1448 | preprotein translocase, YajC subunit | 0.0069 | 0.0049 | 1 | 1.41 |
| HPSNAG_0876 | HPSD74_1100 | periplasmic oligopeptide-binding protein | 0.0069 | 0.0000 | 0.88 | 0 |
| HPSNAG_0158 | HPSD74_0204 | disulfide interchange protein DsbC | 0.0068 | 0.0070 | 0.99 | -1.03 |
| HPSNAG_0920 | HPSD74_1263 | ABC transporter arginine-binding protein | 0.0067 | 0.0053 | 0.95 | 1.27 |
| HPSNAG_0617 | HPSD74_0784 | subtilase family protein | 0.0066 | 0.0073 | 0.96 | -1.11 |
| HPSNAG_1481 | none | ribosomal protein L22 | 0.0066 | 0.0000 | 0 | 0 |
| HPSNAG_1091 | none | putative lipoprotein | 0.0065 | 0.0000 | 0 | 0 |
| HPSNAG_1479 | none | ribosomal protein L16 | 0.0065 | 0.0000 | 0 | 0 |
| HPSNAG_0507 | HPSD74_0762 | ahpC/TSA family protein | 0.0063 | 0.0009 | 1 | 7.22 |
| HPSNAG_1773 | HPSD74_1492 | bamD | 0.0060 | 0.0050 | 0.93 | 1.19 |
| HPSNAG_1132 | HPSD74_1374 | D-alanyl-D-alanine carboxypeptidase dacA | 0.0059 | 0.0023 | 0.99 | 2.57 |
| HPSNAG_1402 | none | ribosomal protein S4 | 0.0059 | 0.0000 | 0 | 0 |
| HPSNAG_2303 | HPSD74_2213 | thioredoxin | 0.0059 | 0.0000 | 0.98 | 0 |
| HPSNAG_0211 | HPSD74_0260 | maltose-binding periplasmic protein | 0.0059 | 0.0018 | 0.99 | 3.28 |
| HPSNAG_1664 | HPSD74_1610 | putative curli production assembly/transport component CsgG | 0.0058 | 0.0041 | 0.96 | 1.40 |
| HPSNAG_0960 | HPSD74_1029 | ubiquinone oxidoreductase, Na(+)-translocating, A subunit | 0.0056 | 0.0028 | 0.91 | 2.02 |
| HPSNAG_0140 | HPSD74_0186 | outer membrane protein P2 | 0.0054 | 0.0114 | 0.75 | -2.11 |
| HPSNAG_1763 | HPSD74_1482 | bacterial extracellular solute-binding s, 5 Middle family protein | 0.0054 | 0.0037 | 0.94 | 1.47 |
| HPSNAG_1815 | HPSD74_0738 | periplasmic binding s and sugar binding domain of LacI family protein | 0.0053 | 0.0042 | 0.88 | 1.26 |
| HPSNAG_2279 | HPSD74_2190 | putative phospholipid-binding lipoprotein mlaA | 0.0052 | 0.0044 | 0.98 | 1.17 |
| HPSNAG_0421 | none | putative lipoprotein | 0.0050 | 0.0000 | 0 | 0 |
| HPSNAG_1386 | HPSD74_2176 | peptidase M13 family protein | 0.0050 | 0.0026 | 0.97 | 1.94 |
| HPSNAG_0041 | HPSD74_0019 | Tol-Pal system beta propeller repeat protein TolB | 0.0050 | 0.0045 | 0.91 | 1.11 |
| HPSNAG_1748 | HPSD74_1465 | lipopolysaccharide-assembly family protein | 0.0047 | 0.0045 | 0.96 | 1.06 |
| HPSNAG_1326 | HPSD74_2040 | ribosomal protein S20 | 0.0047 | 0.0044 | 1 | 1.06 |
| HPSNAG_0216 | HPSD74_0265 | outer membrane lipocarrier protein LolA | 0.0046 | 0.0017 | 0.89 | 2.68 |
| HPSNAG_0366 | HPSD74_0443 | hypothetical protein | 0.0046 | 0.0000 | 1 | 0 |
| HPSNAG_1679 | HPSD74_1625 | periplasmic binding s and sugar binding domain of LacI family protein | 0.0046 | 0.0000 | 0.95 | 0 |
| HPSNAG_1483 | none | ribosomal protein L2 | 0.0045 | 0.0000 | 0 | 0 |
| HPSNAG_2275 | HPSD74_2186 | tail-specific protease | 0.0043 | 0.0026 | 0.98 | 1.65 |
| HPSNAG_1939 | none | hypothetical protein | 0.0042 | 0.0000 | 0 | 0 |
| HPSNAG_1598 | HPSD74_1800 | 2, 3-diketo-L-gulonate-binding periplasmic protein yiaO | 0.0042 | 0.0020 | 0.99 | 2.14 |
| HPSNAG_1663 | HPSD74_1609 | hypothetical protein | 0.0042 | 0.0056 | 1 | -1.33 |
| HPSNAG_1662 | HPSD74_1608 | putative lipoprotein | 0.0042 | 0.0065 | 0.98 | -1.55 |
| HPSNAG_2264 | HPSD74_2159 | hypothetical protein | 0.0041 | 0.0028 | 0.87 | 1.50 |
| HPSNAG_0884 | HPSD74_1108 | hemX family protein | 0.0041 | 0.0026 | 0.93 | 1.58 |
| HPSNAG_0859 | HPSD74_1459 | glucose-specific phosphotransferase enzyme IIA component | 0.0041 | 0.0050 | 0.99 | -1.22 |
| HPSNAG_2056 | HPSD74_1005 | protein QmcA | 0.0040 | 0.0024 | 0.91 | 1.71 |
| HPSNAG_0883 | HPSD74_1107 | hemY family protein | 0.0040 | 0.0018 | 0.96 | 2.20 |
| HPSNAG_2222 | HPSD74_2115 | NAD(P)(+) transhydrogenase (AB-specific), alpha subunit | 0.0039 | 0.0027 | 0.9 | 1.46 |
| HPSNAG_0965 | HPSD74_1034 | ubiquinone oxidoreductase, Na(+)-translocating, F subunit | 0.0039 | 0.0016 | 0.88 | 2.43 |
| HPSNAG_2042 | HPSD74_1917 | chaperonin GroL | 0.0039 | 0.0106 | 0.89 | -2.72 |
| HPSNAG_2091 | none | hep_Hag family protein | 0.0039 | 0.0000 | 0 | 0 |
| HPSNAG_1355 | none | hemoglobin-binding protease hbp autotransporter | 0.0038 | 0.0000 | 0 | 0 |
| HPSNAG_0871 | HPSD74_1447 | hypothetical protein | 0.0037 | 0.0035 | 0.99 | 1.06 |
| HPSNAG_2233 | HPSD74_2126 | transferrin-binding protein 1 | 0.0037 | 0.0048 | 0.84 | -1.29 |
| HPSNAG_1397 | none | ribosomal protein L15 | 0.0037 | 0.0000 | 0 | 0 |
| HPSNAG_1785 | HPSD74_1084 | high-affinity zinc uptake system protein znuA | 0.0036 | 0.0019 | 0.79 | 1.92 |
| HPSNAG_1400 | HPSD74_1566 | 30S ribosomal protein S13 | 0.0036 | 0.0059 | 1 | -1.65 |
| HPSNAG_2232 | HPSD74_2125 | transferrin-binding protein 2 | 0.0036 | 0.0074 | 0.93 | -2.06 |
| HPSNAG_1207 | HPSD74_1510 | hypothetical protein | 0.0034 | 0.0010 | 1 | 3.48 |
| HPSNAG_1633 | HPSD74_1708 | ATP synthase F1, beta subunit | 0.0034 | 0.0025 | 0.96 | 1.36 |
| HPSNAG_1845 | HPSD74_0695 | lipoprotein nlpD | 0.0033 | 0.0037 | 0.84 | -1.10 |
| HPSNAG_0004 | HPSD74_0351 | outer membrane protein P1 | 0.0033 | 0.0068 | 0.81 | -2.07 |
| HPSNAG_0923 | HPSD74_1266 | ribosomal protein L9 | 0.0032 | 0.0028 | 0.8 | 1.13 |
| HPSNAG_1635 | none | ATP synthase F1, alpha subunit | 0.0031 | 0.0000 | 0 | 0 |
| HPSNAG_0997 | HPSD74_1585 | ABC transporter family protein | 0.0031 | 0.0023 | 0.99 | 1.34 |
| HPSNAG_0149 | HPSD74_0195 | hypothetical protein | 0.0031 | 0.0020 | 0.93 | 1.56 |
| HPSNAG_1359 | none | anaerobic ribonucleotide reductase activating NrdG domain protein | 0.0029 | 0.0000 | 0 | 0 |
| HPSNAG_2134 | HPSD74_0356 | efflux transporter, RND family, MFP subunit | 0.0029 | 0.0000 | 0.94 | 0 |
| HPSNAG_0492 | HPSD74_0593 | dihydrolipoyllysine-residue acetyltransferase | 0.0029 | 0.0024 | 0.94 | 1.21 |
| HPSNAG_0492 | none | dihydrolipoyllysine-residue acetyltransferase | 0.0029 | 0.0000 | 0 | 0 |
| HPSNAG_1324 | HPSD74_2038 | cysteine synthase A | 0.0029 | 0.0029 | 0.85 | -1.02 |
| HPSNAG_1016 | HPSD74_1069 | PPIC-type PPIASE domain protein | 0.0029 | 0.0013 | 0.9 | 2.20 |
| HPSNAG_0012 | none | glycosyl transferase 2 family protein | 0.0029 | 0.0000 | 0 | 0 |
| HPSNAG_0251 | none | translation elongation factor Ts | 0.0028 | 0.0000 | 0 | 0 |
| HPSNAG_0038 | HPSD74_0016 | protein TolQ | 0.0028 | 0.0011 | 0.85 | 2.58 |
| HPSNAG_1480 | none | ribosomal protein S3 | 0.0027 | 0.0000 | 0 | 0 |
| HPSNAG_0039 | HPSD74_0017 | protein tolR | 0.0027 | 0.0021 | 0.93 | 1.26 |
| HPSNAG_2140 | HPSD74_2247 | S-adenosyl-l-methionine hydroxide adenosyltransferase family protein | 0.0027 | 0.0046 | 0.99 | -1.71 |
| HPSNAG_0442 | HPSD74_0537 | D-methionine-binding lipoprotein metQ | 0.0027 | 0.0011 | 1 | 2.33 |
| HPSNAG_0194 | HPSD74_0242 | hypothetical protein | 0.0026 | 0.0000 | 0.97 | 0 |
| HPSNAG_2117 | HPSD74_1951 | type IV pilus biogenesis/stability protein PilW | 0.0026 | 0.0014 | 1 | 1.90 |
| HPSNAG_1590 | HPSD74_1787 | disulfide interchange protein DsbA | 0.0026 | 0.0028 | 1 | -1.06 |
| HPSNAG_1906 | none | ribosomal protein L20 | 0.0026 | 0.0000 | 0 | 0 |
| HPSNAG_2041 | none | 10 kDa chaperonin | 0.0025 | 0.0000 | 0 | 0 |
| HPSNAG_0491 | HPSD74_0592 | dihydrolipoyl dehydrogenase | 0.0025 | 0.0028 | 0.92 | -1.09 |
| HPSNAG_0851 | HPSD74_1210 | 3-oxoacyl-[acyl-carrier-protein] reductase | 0.0025 | 0.0033 | 0.95 | -1.33 |
| HPSNAG_1503 | HPSD74_0770 | rhodanese-like domain protein | 0.0025 | 0.0017 | 0.97 | 1.44 |
| HPSNAG_2220 | HPSD74_2113 | cytochrome c-type biogenesis protein CcmE | 0.0024 | 0.0022 | 1 | 1.11 |
| HPSNAG_0962 | HPSD74_1031 | ubiquinone oxidoreductase, Na(+)-translocating, C subunit | 0.0024 | 0.0023 | 0.93 | 1.03 |
| HPSNAG_0727 | HPSD74_0870 | outer membrane autotransporter barrel domain protein (aidA) | 0.0024 | 0.0222 | 0.68 | -9.40 |
| HPSNAG_0644 | HPSD74_0845 | hypothetical protein | 0.0024 | 0.0013 | 0.91 | 1.78 |
| HPSNAG_1272 | HPSD74_1314 | putative thiol peroxidase | 0.0023 | 0.0013 | 0.99 | 1.69 |
| HPSNAG_2247 | HPSD74_2141 | catalase | 0.0023 | 0.0020 | 1 | 1.16 |
| HPSNAG_0475 | HPSD74_0572 | polyribonucleotide nucleotidyltransferase | 0.0022 | 0.0015 | 0.95 | 1.52 |
| HPSNAG_2098 | none | helix-turn-helix family protein | 0.0022 | 0.0000 | 0 | 0 |
| HPSNAG_0138 | HPSD74_0183 | ribosomal protein L13 | 0.0022 | 0.0033 | 1 | -1.51 |
| HPSNAG_1820 | HPSD74_0733 | PTS system, mannose/fructose/sorbose , IID component family protein | 0.0022 | 0.0015 | 0.81 | 1.50 |
| HPSNAG_2221 | HPSD74_2114 | NAD(P) transhydrogenase subunit beta | 0.0022 | 0.0013 | 0.93 | 1.66 |
| HPSNAG_1133 | HPSD74_1375 | rare lipoA family protein | 0.0022 | 0.0030 | 0.85 | -1.37 |
| HPSNAG_0793 | HPSD74_0969 | co-chaperone HscB | 0.0021 | 0.0023 | 0.92 | -1.05 |
| HPSNAG_0459 | HPSD74_0553 | ribosomal protein S1 | 0.0021 | 0.0016 | 0.9 | 1.36 |
| HPSNAG_0239 | HPSD74_0361 | cytolethal distending toxin subunit A | 0.0021 | 0.0084 | 0.9 | -3.96 |
| HPSNAG_1393 | none | ribosomal protein L6 | 0.0021 | 0.0000 | 0 | 0 |
| HPSNAG_1401 | HPSD74_1565 | 30S ribosomal protein S11 | 0.0021 | 0.0044 | 1 | -2.08 |
| HPSNAG_0946 | HPSD74_1246 | hypothetical protein | 0.0021 | 0.0026 | 0.94 | -1.23 |
| HPSNAG_0034 | HPSD74_0012 | cytochrome d ubiquinol oxidase subunit 1 | 0.0020 | 0.0010 | 0.94 | 2.06 |
| HPSNAG_1877 | HPSD74_2005 | TRAP transporter solute receptor, TAXI family protein | 0.0020 | 0.0047 | 0.94 | -2.33 |
| HPSNAG_0101 | HPSD74_0075 | signal peptidase I | 0.0020 | 0.0015 | 0.97 | 1.31 |
| HPSNAG_0493 | HPSD74_0594 | pyruvate dehydrogenase (acetyl-transferring), homodimeric type | 0.0020 | 0.0022 | 0.99 | -1.12 |
| HPSNAG_0725 | HPSD74_0868 | DNA protection during starvation protein | 0.0020 | 0.0027 | 1 | -1.33 |
| HPSNAG_0020 | HPSD74_0331 | cysteine desulfurase IscS | 0.0019 | 0.0019 | 0.97 | -1.00 |
| HPSNAG_0700 | HPSD74_0087 | hypothetical protein | 0.0019 | 0.0010 | 0.86 | 1.85 |
| HPSNAG_2108 | HPSD74_1942 | fructose-bisphosphate aldolase, class II | 0.0019 | 0.0023 | 0.95 | -1.21 |
| HPSNAG_0018 | HPSD74_0333 | tyrosine-protein kinase etk | 0.0019 | 0.0004 | 0.95 | 4.42 |
| HPSNAG_1376 | HPSD74_1556 | 50S ribosomal protein L10 | 0.0019 | 0.0028 | 1 | -1.47 |
| HPSNAG_0711 | HPSD74_0854 | surA N-terminal domain protein | 0.0019 | 0.0000 | 0.98 | 0 |
| HPSNAG_1085 | HPSD74_1847 | D-lactate dehydrogenase, membrane binding family protein | 0.0019 | 0.0008 | 0.99 | 2.40 |
| HPSNAG_1085 | none | D-lactate dehydrogenase, membrane binding family protein | 0.0019 | 0.0000 | 0 | 0 |
| HPSNAG_0335 | HPSD74_0406 | hypothetical protein | 0.0018 | 0.0020 | 0.96 | -1.11 |
| HPSNAG_0074 | HPSD74_0050 | apbE family protein | 0.0018 | 0.0025 | 0.99 | -1.43 |
| HPSNAG_0874 | HPSD74_1097 | proton antiporter-2 family protein | 0.0016 | 0.0004 | 0.9 | 3.89 |
| HPSNAG_0789 | HPSD74_0965 | spermidine/putrescine import ATP-binding protein PotA | 0.0016 | 0.0009 | 0.99 | 1.76 |
| HPSNAG_0509 | HPSD74_0756 | peptide methionine sulfoxide reductase MsrA/MsrB | 0.0016 | 0.0000 | 1 | 0 |
| HPSNAG_1930 | HPSD74_2170 | translation elongation factor G | 0.0016 | 0.0019 | 0.95 | -1.19 |
| HPSNAG_0339 | none | collagen triple helix repeat family protein | 0.0016 | 0.0000 | 0 | 0 |
| HPSNAG_2177 | HPSD74_2063 | efflux transporter, outer membrane factor (OMF) lipo, NodT family protein | 0.0016 | 0.0021 | 0.89 | -1.30 |
| HPSNAG_0806 | HPSD74_0979 | protease HtpX | 0.0016 | 0.0009 | 0.95 | 1.67 |
| HPSNAG_2293 | HPSD74_2203 | penicillin-binding protein 1A | 0.0015 | 0.0007 | 0.97 | 2.12 |
| HPSNAG_2022 | HPSD74_1908 | outer membrane lipoprotein LolB | 0.0015 | 0.0035 | 0.87 | -2.39 |
| HPSNAG_1337 | HPSD74_1971 | cell division protein FtsN | 0.0014 | 0.0019 | 0.82 | -1.35 |
| HPSNAG_1823 | HPSD74_0730 | ferrochelatase | 0.0014 | 0.0000 | 0.9 | 0 |
| HPSNAG_1725 | HPSD74_1695 | DNA-directed RNA polymerase, beta' subunit | 0.0014 | 0.0046 | 0.9 | -3.35 |
| HPSNAG_2168 | HPSD74_2052 | 3-oxoacyl-[acyl-carrier-protein] synthase 1 | 0.0014 | 0.0014 | 0.9 | -1.01 |
| HPSNAG_0181 | none | collagen triple helix repeat family protein | 0.0014 | 0.0000 | 0 | 0 |
| HPSNAG_0756 | HPSD74_0667 | enoyl-[acyl-carrier-protein] reductase | 0.0013 | 0.0017 | 1 | -1.25 |
| HPSNAG_0729 | HPSD74_0872 | putative 2-hydroxyacid dehydrogenase | 0.0013 | 0.0008 | 0.9 | 1.59 |
| HPSNAG_0869 | HPSD74_1449 | protein-export membrane protein SecD | 0.0013 | 0.0009 | 0.98 | 1.37 |
| HPSNAG_1864 | HPSD74_2016 | chaperone protein DnaK | 0.0013 | 0.0028 | 0.89 | -2.17 |
| HPSNAG_1217 | HPSD74_1520 | peptidase M48 family protein | 0.0013 | 0.0014 | 0.93 | -1.08 |
| HPSNAG_2228 | HPSD74_2121 | hypothetical protein | 0.0012 | 0.0006 | 0.97 | 1.94 |
| HPSNAG_0238 | HPSD74_0360 | cytolethal distending toxin protein B | 0.0012 | 0.0122 | 0.93 | -10.32 |
| HPSNAG_2163 | HPSD74_2223 | fumarate reductase, flavoprotein subunit | 0.0012 | 0.0008 | 0.92 | 1.54 |
| HPSNAG_2069 | HPSD74_1017 | NADH dehydrogenase | 0.0012 | 0.0008 | 0.97 | 1.52 |
| HPSNAG_0097 | HPSD74_0071 | cell division protein FtsZ | 0.0012 | 0.0007 | 0.85 | 1.67 |
| HPSNAG_2310 | HPSD74_2219 | glyceraldehyde-3-phosphate dehydrogenase, type I | 0.0010 | 0.0010 | 0.91 | 1.01 |
| HPSNAG_0640 | HPSD74_0841 | hypothetical protein | 0.0010 | 0.0009 | 0.97 | 1.11 |
| HPSNAG_2244 | HPSD74_2138 | phosphopyruvate hydratase | 0.0010 | 0.0019 | 0.96 | -1.90 |
| HPSNAG_1150 | HPSD74_1391 | thiamin/thiamine pyrophosphate ABC transporter, thiamin/thiamine pyrophospate-binding protein | 0.0010 | 0.0020 | 0.91 | -2.06 |
| HPSNAG_1343 | HPSD74_1977 | putative protease sohB | 0.0010 | 0.0004 | 0.88 | 2.28 |
| HPSNAG_1319 | HPSD74_2033 | bacterial extracellular solute-binding family protein | 0.0010 | 0.0014 | 0.99 | -1.40 |
| HPSNAG_1167 | HPSD74_1408 | bifunctional aspartokinase/homoserine dehydrogenase 1 | 0.0009 | 0.0005 | 0.94 | 1.93 |
| HPSNAG_1723 | HPSD74_1694 | DNA-directed RNA polymerase, beta subunit | 0.0009 | 0.0027 | 0.93 | -2.89 |
| HPSNAG_1537 | none | phage T7 tail fiber family protein | 0.0009 | 0.0000 | 0 | 0 |
| HPSNAG_0650 | HPSD74_0129 | transporter, lactate permease family protein | 0.0009 | 0.0003 | 0.99 | 2.94 |
| HPSNAG_2024 | HPSD74_1910 | ribose-phosphate pyrophosphokinase | 0.0009 | 0.0009 | 1 | -1.04 |
| HPSNAG_2148 | HPSD74_2239 | glycerol-3-phosphate O-acyltransferase | 0.0009 | 0.0011 | 0.98 | -1.26 |
| HPSNAG_2224 | HPSD74_2117 | rod shape-determining protein mreB | 0.0008 | 0.0000 | 1 | 0 |
| HPSNAG_0338 | none | phage T7 tail fiber family protein | 0.0008 | 0.0000 | 0 | 0 |
| HPSNAG_0933 | HPSD74_1233 | transaldolase | 0.0008 | 0.0034 | 0.99 | -4.25 |
| HPSNAG_0040 | none | protein TolA | 0.0008 | 0.0000 | 0 | 0 |
| HPSNAG_1352 | HPSD74_1531 | outer membrane autotransporter barrel domain protein | 0.0008 | 0.0020 | 0.91 | -2.51 |
| HPSNAG_0296 | none | cytolethal distending toxin protein B | 0.0007 | 0.0000 | 0 | 0 |
| HPSNAG_0473 | HPSD74_0569 | cold-shock DEAD box protein A | 0.0007 | 0.0031 | 0.89 | -4.65 |
| HPSNAG_1092 | HPSD74_1168 | ribonuclease E | 0.0006 | 0.0010 | 0.87 | -1.69 |
| HPSNAG_1078 | HPSD74_1181 | protein RecA | 0.0005 | 0.0016 | 0 | -3.58 |
| HPSNAG_0365 | HPSD74_0442 | phosphate acetyltransferase | 0.0004 | 0.0012 | 0.98 | -2.83 |
| HPSNAG_2312 | none | phage T7 tail fiber family protein | 0.0003 | 0.0000 | 0 | 0 |
| HPSNAG_1250 | HPSD74_0874 | transposase, Mutator family protein | 0.0003 | 0.0065 | 1 | -22.54 |
| HPSNAG_1315 | HPSD74_2030 | delta-1-pyrroline-5-carboxylate dehydrogenase | 0.0003 | 0.0004 | 0.97 | -1.31 |
| none | HPSD74_1658 | outer membrane autotransporter barrel domain protein | 0.0000 | 0.0145 | 0 | 0 |
| none | HPSD74_1715 | subtilase family protein | 0.0000 | 0.0099 | 0 | 0 |
| HPSNAG_1517 | HPSD74_1581 | hypothetical protein | 0.0000 | 0.0079 | 0.86 | 0 |
| HPSNAG_2260 | HPSD74_2155 | phosphate/phosphite/phosphonate ABC transporter, periplasmic binding family protein | 0.0000 | 0.0078 | 0.88 | 0 |
| none | HPSD74_1347 | outer membrane lipoprotein pcp | 0.0000 | 0.0076 | 0 | 0 |
| none | HPSD74_1526 | hypothetical protein | 0.0000 | 0.0075 | 0 | 0 |
| HPSNAG_0122 | HPSD74_0166 | outer membrane autotransporter barrel domain protein | 0.0000 | 0.0061 | 0.79 | 0 |
| HPSNAG_0021 | HPSD74_0330 | scaffold protein involved in iron-sulfur cluster assembly | 0.0000 | 0.0059 | 1 | 0 |
| none | HPSD74_0280 | hypothetical protein | 0.0000 | 0.0055 | 0 | 0 |
| none | HPSD74_1714 | autotransporter beta-domain protein | 0.0000 | 0.0053 | 0 | 0 |
| HPSNAG_1258 | HPSD74_1299 | ribosomal protein L21 | 0.0000 | 0.0052 | 0.84 | 0 |
| none | HPSD74_1529 | hypothetical protein | 0.0000 | 0.0052 | 0 | 0 |
| HPSNAG_1929 | HPSD74_2171 | ribosomal protein S7 | 0.0000 | 0.0051 | 1 | 0 |
| HPSNAG_1316 | HPSD74_2031 | ribonucleotide reductase, small chain family protein | 0.0000 | 0.0049 | 0.95 | 0 |
| none | HPSD74_1169 | hypothetical protein | 0.0000 | 0.0047 | 0.9 | 0 |
| HPSNAG_0926 | HPSD74_1269 | ribosomal protein S6 | 0.0000 | 0.0041 | 0.88 | 0 |
| HPSNAG_1214 | HPSD74_1517 | glycerophosphoryl diester phosphodiesterase | 0.0000 | 0.0036 | 0.96 | 0 |
| none | HPSD74_2222 | bifunctional riboflavin kinase/FMN adenylyltransferase | 0.0000 | 0.0034 | 0 | 0 |
| HPSNAG_0929 | HPSD74_1272 | hypothetical protein | 0.0000 | 0.0034 | 0.99 | 0 |
| HPSNAG_1784 | HPSD74_1085 | CDP-diacylglycerol--serine O-phosphatidyltransferase | 0.0000 | 0.0034 | 0.98 | 0 |
| none | HPSD74_1865 | class B acid phosphatase | 0.0000 | 0.0034 | 0 | 0 |
| HPSNAG_0664 | HPSD74_0117 | integration host factor, alpha subunit | 0.0000 | 0.0031 | 0.99 | 0 |
| none | HPSD74_2260 | periplasmic oligopeptide-binding domain protein | 0.0000 | 0.0031 | 0 | 0 |
| HPSNAG_0861 | HPSD74_1457 | hypothetical protein | 0.0000 | 0.0030 | 0.99 | 0 |
| none | HPSD74_0579 | tonB-dependent siderophore receptor family protein | 0.0000 | 0.0029 | 0 | 0 |
| HPSNAG_0139 | HPSD74_0184 | 30S ribosomal subunit protein S9 | 0.0000 | 0.0029 | 1 | 0 |
| none | HPSD74_1707 | ATP synthase family protein | 0.0000 | 0.0029 | 0 | 0 |
| HPSNAG_0257 | HPSD74_0285 | hypothetical protein | 0.0000 | 0.0028 | 0.94 | 0 |
| HPSNAG_0672 | HPSD74_0109 | hypothetical protein | 0.0000 | 0.0027 | 0.99 | 0 |
| none | HPSD74_1916 | tonB-dependent Receptor Plug domain protein | 0.0000 | 0.0027 | 0 | 0 |
| none | HPSD74_1534 | hypothetical protein | 0.0000 | 0.0022 | 0 | 0 |
| HPSNAG_1777 | HPSD74_1092 | phosphoribosylformylglycinamidine cyclo-ligase | 0.0000 | 0.0022 | 0.96 | 0 |
| HPSNAG_0112 | HPSD74_0083 | ribosome recycling factor | 0.0000 | 0.0021 | 0.99 | 0 |
| HPSNAG_0157 | HPSD74_0203 | ATP-dependent chaperone ClpB | 0.0000 | 0.0021 | 0.96 | 0 |
| none | HPSD74_1809 | ribosomal protein L1 | 0.0000 | 0.0020 | 0 | 0 |
| HPSNAG_0657 | HPSD74_1884 | pseudouridine synthase family protein | 0.0000 | 0.0020 | 1 | 0 |
| none | HPSD74_0044 | tonB-dependent siderophore receptor family protein | 0.0000 | 0.0020 | 0 | 0 |
| HPSNAG_1509 | HPSD74_1740 | nicotinate phosphoribosyltransferase | 0.0000 | 0.0019 | 0.91 | 0 |
| none | HPSD74_0814 | filamentous hemagglutinin family N-terminal domain protein | 0.0000 | 0.0018 | 0 | 0 |
| HPSNAG_1935 | HPSD74_1862 | nicotinamide-nucleotide adenylyltransferase | 0.0000 | 0.0018 | 0.92 | 0 |
| none | HPSD74_0436 | filamentous hemagglutinin family N-terminal domain protein | 0.0000 | 0.0018 | 0 | 0 |
| HPSNAG_2319 | HPSD74_1446 | argininosuccinate synthase | 0.0000 | 0.0018 | 0.98 | 0 |
| HPSNAG_0892 | HPSD74_1117 | ATP-dependent protease La | 0.0000 | 0.0018 | 0.95 | 0 |
| HPSNAG_1173 | HPSD74_1414 | 2', 3'-cyclic-nucleotide 2'-phosphodiesterase | 0.0000 | 0.0017 | 0.91 | 0 |
| none | HPSD74_1544 | inner-membrane protein insertion factor | 0.0000 | 0.0016 | 0 | 0 |
| HPSNAG_2285 | HPSD74_2195 | isocitrate dehydrogenase, NADP-dependent | 0.0000 | 0.0016 | 0.95 | 0 |
| HPSNAG_0056 | HPSD74_0034 | lipoprotein HlpB, chromosome segregation ATPase | 0.0000 | 0.0016 | 0.98 | 0 |
| HPSNAG_0264 | HPSD74_0293 | inosine-5'-monophosphate dehydrogenase | 0.0000 | 0.0015 | 0.94 | 0 |
| HPSNAG_0944 | HPSD74_1244 | FKBP-type peptidyl-prolyl cis-trans isomerase slyD | 0.0000 | 0.0015 | 0.74 | 0 |
| HPSNAG_0351 | HPSD74_0422 | glyA | 0.0000 | 0.0015 | 0.95 | 0 |
| HPSNAG_1602 | HPSD74_1817 | transcription termination/antitermination factor NusG | 0.0000 | 0.0015 | 0.99 | 0 |
| HPSNAG_1534 | HPSD74_1589 | signal recognition particle protein | 0.0000 | 0.0015 | 0.89 | 0 |
| HPSNAG_1765 | HPSD74_1484 | deoxyguanosinetriphosphate triphosphohydrolase, family protein | 0.0000 | 0.0014 | 0.98 | 0 |
| HPSNAG_1463 | HPSD74_1576 | molybdate ABC transporter, periplasmic molybdate-binding protein | 0.0000 | 0.0014 | 0.81 | 0 |
| none | HPSD74_1277 | N-6 DNA Methylase family protein | 0.0000 | 0.0014 | 0 | 0 |
| none | HPSD74_1356 | hypothetical protein | 0.0000 | 0.0013 | 0 | 0 |
| none | HPSD74_1440 | transketolase | 0.0000 | 0.0013 | 0 | 0 |
| HPSNAG_1182 | HPSD74_1424 | acetyl-CoA carboxylase, biotin carboxylase | 0.0000 | 0.0012 | 0.98 | 0 |
| HPSNAG_1690 | HPSD74_1637 | MTA/SAH nucleosidase | 0.0000 | 0.0012 | 0.99 | 0 |
| HPSNAG_0080 | HPSD74_0056 | putative inositol-1-monophosphatase | 0.0000 | 0.0012 | 0.93 | 0 |
| none | HPSD74_0885 | gamma-glutamyltransferase | 0.0000 | 0.0012 | 0 | 0 |
| HPSNAG_1183 | HPSD74_1426 | acetyl-CoA carboxylase, biotin carboxyl carrier protein | 0.0000 | 0.0011 | 0.72 | 0 |
| HPSNAG_0941 | HPSD74_1241 | ribose 5-phosphate isomerase A | 0.0000 | 0.0011 | 0.99 | 0 |
| none | HPSD74_2044 | putative tonB-dependent receptor | 0.0000 | 0.0010 | 0 | 0 |
| HPSNAG_0896 | HPSD74_1121 | uracil phosphoribosyltransferase | 0.0000 | 0.0010 | 1 | 0 |
| HPSNAG_1461 | HPSD74_1574 | bacterial extracellular solute-binding s, 5 Middle family protein | 0.0000 | 0.0009 | 0.96 | 0 |
| none | HPSD74_1471 | type I secretion membrane fusion , HlyD family protein | 0.0000 | 0.0009 | 0 | 0 |
| none | HPSD74_1336 | hisB | 0.0000 | 0.0009 | 0 | 0 |
| HPSNAG_2191 | HPSD74_2081 | pyruvate kinase | 0.0000 | 0.0009 | 0.91 | 0 |
| none | HPSD74_1933 | lipid A export permease/ATP-binding protein MsbA | 0.0000 | 0.0009 | 0 | 0 |
| none | HPSD74_1115 | ngoPII restriction endonuclease family protein | 0.0000 | 0.0009 | 0 | 0 |
| HPSNAG_0709 | HPSD74_0852 | small GTP-binding domain protein | 0.0000 | 0.0009 | 0.92 | 0 |
| none | HPSD74_0813 | hemolysin secretion/activation ShlB/FhaC/HecB family protein | 0.0000 | 0.0008 | 0 | 0 |
| none | HPSD74_0887 | hemolysin secretion/activation ShlB/FhaC/HecB family protein | 0.0000 | 0.0008 | 0 | 0 |
| HPSNAG_0846 | HPSD74_1215 | putative phosphomannomutase | 0.0000 | 0.0008 | 0.99 | 0 |
| HPSNAG_0940 | HPSD74_1240 | serA | 0.0000 | 0.0007 | 0.99 | 0 |
| HPSNAG_0043 | HPSD74_0021 | dnaJ domain protein | 0.0000 | 0.0007 | 0.82 | 0 |
| none | HPSD74_0018 | protein TolA | 0.0000 | 0.0007 | 0 | 0 |
| HPSNAG_1088 | HPSD74_1172 | sigma70 factor RNA polymerase, sigma 70 (sigma D) factor | 0.0000 | 0.0006 | 0.78 | 0 |
| HPSNAG_0344 | HPSD74_0413 | single-stranded DNA-binding protein | 0.0000 | 0.0006 | 0.79 | 0 |
| HPSNAG_0761 | HPSD74_0681 | membrane-bound lytic murein transglycosylase A | 0.0000 | 0.0005 | 1 | 0 |
| HPSNAG_1781 | HPSD74_1088 | isoleucyl-tRNA synthetase | 0.0000 | 0.0004 | 0.99 | 0 |
| HPSNAG_1578 | HPSD74_1775 | periplasmic nitrate reductase, large subunit | 0.0000 | 0.0004 | 0.97 | 0 |
| HPSNAG_0449 | HPSD74_0544 | putative YadA-like protein | 0.0000 | 0.0003 | 0.96 | 0 |
| none | HPSD74_0993 | hypothetical protein | 0.0000 | 0.0001 | 0 | 0 |
